# Enabling target-aware molecule generation to follow multi objectives with Pareto MCTS

**DOI:** 10.1038/s42003-024-06746-w

**Published:** 2024-09-02

**Authors:** Yaodong Yang, Guangyong Chen, Jinpeng Li, Junyou Li, Odin Zhang, Xujun Zhang, Lanqing Li, Jianye Hao, Ercheng Wang, Pheng-Ann Heng

**Affiliations:** 1 Department of Computer Science and Engineering, The Chinese University of Hong Kong, Hong Kong, China; 2 Zhejiang Lab, Hangzhou, China; 3 Zhejiang University, Hangzhou, China; 4 Noah’s Ark Lab, Huawei, Shenzhen, China

**Keywords:** Pharmaceutics, Computational models

## Abstract

Target-aware drug discovery has greatly accelerated the drug discovery process to design small-molecule ligands with high binding affinity to disease-related protein targets. Conditioned on targeted proteins, previous works utilize various kinds of deep generative models and have shown great potential in generating molecules with strong protein-ligand binding interactions. However, beyond binding affinity, effective drug molecules must manifest other essential properties such as high drug-likeness, which are not explicitly addressed by current target-aware generative methods. In this article, aiming to bridge the gap of multi-objective target-aware molecule generation in the field of deep learning-based drug discovery, we propose ParetoDrug, a Pareto Monte Carlo Tree Search (MCTS) generation algorithm. ParetoDrug searches molecules on the Pareto Front in chemical space using MCTS to enable synchronous optimization of multiple properties. Specifically, ParetoDrug utilizes pretrained atom-by-atom autoregressive generative models for the exploration guidance to desired molecules during MCTS searching. Besides, when selecting the next atom symbol, a scheme named ParetoPUCT is proposed to balance exploration and exploitation. Benchmark experiments and case studies demonstrate that ParetoDrug is highly effective in traversing the large and complex chemical space to discover novel compounds with satisfactory binding affinities and drug-like properties for various multi-objective target-aware drug discovery tasks.

## Abstract (web summary)

A multi-objective molecule generation method with Pareto MCTS as searching procedure in chemical space and the pretrained molecule generative model conditioned on protein targets as searching guidance to synchronously optimize multiple properties.

## Introduction

The rational design of molecules to act as clinical drugs remains a significant challenge in biopharmaceutical research, especially concerning the attainment of favorable physiochemical and pharmacological properties. In support of such endeavors, target-based drug discovery aims to identify small-molecule ligands that exhibit high affinity and specificity for a particular protein pocket structure^1^. Traditionally, target-based drug discovery has been approached through either high-throughput experimental methods or virtual screening of extensive chemical databases^2,3^ targeted at specific biomolecular targets^4,5^. Subsequently, the screening of bioanalytical indicators through elaborate clinical experiments is conducted to evaluate drug-like properties. This pursuit contributes to the conventional 10-year drug development cycle and staggering research and development costs of approximately 2.8 billion USD, coupled with a remarkably high failure rate. The predetermined selection of compounds for screening further constrains the exploration of chemical space, tethering it to historical knowledge derived from previously investigated molecules. This ultimately leads to a fervent industry focus on popular drug targets, resulting in the challenge that the molecules selected through screening are unable to avoid patent restrictions. In contrast, recent advancements in target-aware molecule generation, particularly the development of generative models trained on extensive datasets, present a promising paradigm shift. These models, rooted in deep learning, offer an innovative approach to expedite ligand discovery and optimization. They achieve this by generating entirely novel and diverse molecules capable of binding to a specified protein target, starting from scratch^6^. This transformative approach holds great potential to overcome the limitations associated with traditional methods, offering a more efficient and expansive exploration of the entire chemical space.

Since the first inception of an autoencoder model conditioned on targeted proteins in 2018^7^, there has been rapid progress in deep learning-based target-aware molecule generation methods. Various works take advantage of conditional generative models, such as the autoencoder^7–9^, generative adversarial network^10^, and diffusion model^11,12^, to infer entire molecules through a one-time feedforward process, incorporating binding site information as input. Moreover, to enhance structural representation, convolutional neural networks^7^ and graph convolutional networks^8^ are employed. In the meantime, some approaches utilize voxelized representations^10^ or atomic density grids^13^ to characterize compound-receptor complexes. Another pivotal category of deep learning-based target-aware drug discovery involves autoregressive generative models, which predict the next atom (and its position) sequentially conditioned on the molecular fragment and binding site information. To model the conditioned intermediate context, diverse network architectures like transformers^14,15^, recurrent neural networks^16,17^, and flow models^18^ are introduced as the context encoder. Additionally, graph neural networks^18–20^ are widely utilized to extract chemical and geometrical features of ligands and protein pockets. However, these efforts are not yet integrated into mainstream drug discovery practices, and a significant obstacle lies in the inherent multi-objective optimization nature of drug discovery ^21^. Beyond strong binding affinity to the targeted protein, drug molecules must exhibit other desirable properties, such as high drug-likeness and low toxicity. Presently, existing deep learning-based target-aware generative methods predominantly focus on the single objective of optimizing binding affinity. The multi-objective nature of drug molecules, with sometimes conflicting demands, necessitates ongoing development of novel multi-objective target-aware drug discovery techniques to enhance the overall success rates of drug discovery.

Conversely, numerous studies have explored the domain of general multi-objective drug discovery. Certain approaches, such as MolGPT^22^, fall within the ligand-based methodology, aspiring to generate novel compounds with favorable physicochemical properties. However, these methods fall short in incorporating protein information, thus lacking assurance that the generated molecules can effectively bind to specified protein targets. Concurrently, other methodologies like MCMG^21^, RationaleRL^23^, MolSearch^24^, and GENERA^25^ aim to optimize not only the binding affinity objective but also other property objectives. Specifically, these methodologies leverage optimization techniques such as reinforcement learning^26^ and genetic algorithms^27^ to enhance the binding affinity objective predicted by machine learning-based or simulation-based docking score functions. However, a notable drawback is their failure to explicitly incorporate target protein information when constructing generative models. The absence of protein information renders the optimization of the binding affinity objective inefficient, and the resulting generative models from these target-scoring-based methods cannot be readily generalized to other protein targets. In contrast to ligand-based and target-scoring-based approaches, a recent development is CProMG^28^, designed to generate molecules that meet multiple property constraints with an enhanced representation of protein structure information. CProMG treats this task as a multi-constraint molecule generation problem, with each property constraint set to exceed a predefined threshold. However, CProMG does not attempt to maximize molecule properties through optimization techniques for a comprehensive exploration of the chemical space. A more in-depth discussion is provided in the Discussion section.

Similar challenges also exist in natural language generation tasks, where models predicting the next token often express unintended behaviors, such as making up facts, generating biased or toxic text, or not following user instructions. To address this issue, OpenAI focuses on fine-tuning approaches to align language models. Specifically, they employ reinforcement learning from human feedback (RLHF) to fine-tune GPT-3^29^ to follow a broad class of written instructions^30^. In contrast to the fuzzy, hard-to-quantify human values in natural language tasks, we can explicitly calculate multiple molecular metrics in the context of drug development.

In this study, we explore the use of an autoregressive Pareto Monte Carlo Tree Search (MCTS) generation algorithm named ParetoDrug for the design of drug molecules to address the existing gap in multi-objective target-aware drug discovery within the domain of deep learning-based drug discovery. This algorithm effectively facilitates the simultaneous optimization of multiple molecule properties. In its operation, ParetoDrug first explores molecules on the Pareto Front within the chemical space. It achieves this by maintaining a global pool comprising Pareto optimal molecules, each of which is not surpassed by another molecule in the same pool across every property objective. During the exploration process, ParetoDrug leverages existing pretrained autoregressive target-aware molecule generation models to guide the search for the next atom symbol, facilitating the identification of molecules with high binding affinity to protein targets. Additionally, in the selection of the next atom symbol, ParetoDrug introduces a scheme named ParetoPUCT. This scheme is designed to balance the exploration of chemical space and the exploitation of the pretrained autoregressive generative model. Through these strategies, ParetoDrug owns the ability to generate molecules with multiple desirable properties, including binding affinity. Computational evaluations on the benchmark dataset and case studies, including multi-objective target-aware drug discovery tasks for known drugs (e.g., Tropifexor and Copanlisib), a multi-target drug discovery task for HIV-related disease targets, and a multi-target multi-objective drug discovery task for a dual-inhibitor Lapatinib, demonstrate the high effectiveness of ParetoDrug. The algorithm exhibits proficiency in discovering small-molecule drug candidates possessing multiple required properties, particularly including binding affinities to specified protein targets.

## Results

In this section, we first conduct the experiments on a benchmark to demonstrate ParetoDrug’s remarkable ability to generate molecules with multiple desired properties including the binding affinity and drug-like properties when compared with various baselines. Meanwhile, we also give the statistical analysis of the generated molecules of ParetoDrug. Then we use ParetoDrug to perform the case studies for the multi-objective target-aware drug discovery task, multi-target drug discovery task, and multi-target multi-objective drug discovery task respectively. In these case studies, ParetoDrug is able to generate the Pareto Dominate molecules over the known drug ligands in terms of the specified molecule property objectives, which exhibits the promising molecule discovery potential of ParetoDrug.

### Benchmark experiments

In the benchmark experiments, we follow the settings as Qian et al.^15^ where there are 100 protein targets sampled from the public database of protein-ligand pairs BindingDB^31^ as the test set. For each test protein target, we generate 10 candidate molecules for evaluation. All 1000 candidate molecules are evaluated by a set of molecule property metrics, and the scores are averaged for an overall comparison. Please refer to Supplementary Information A and B for a detailed experimental and hyperparameter setup. We use several important metrics to evaluate the generated molecules, including docking score, uniqueness, LogP, QED, SA score, and NP-likeness described as follows.

- **Docking score**. Binding energy is regarded as a general indicator to describe the binding affinity between molecule ligands and target proteins. Specifically, we utilize a free and widely used tool called smina^32^ to compute the binding affinity. We use the negative value of the output by smina as the docking score. The higher the docking score is, the better the molecule is docked into the target protein.
- **Uniqueness**. Drug design models should be able to generate different molecules conditioning on different target proteins. The higher the uniqueness value is, the more sensitive the model is to the specified target protein. This metric is computed as follows:
  1 $${{{{\rm{Uniqueness}}}}}( \% )=\frac{\#({{{{\rm{Set}}}}}({\cup }_{{S}_{{{{{\rm{p}}}}}}\in {{\mathbb{S}}}_{{{{{\rm{p}}}}}}}{{{{\rm{Set}}}}}({M}_{{s}_{{{{{\rm{p}}}}}}})))}{\#({\cup }_{{S}_{{{{{\rm{p}}}}}}\in {{\mathbb{S}}}_{{{{{\rm{p}}}}}}}\,{{{{\rm{Set}}}}}({M}_{{S}_{{{{{\rm{p}}}}}}}))}\times 100 \% ,$$
  where ${{\mathbb{S}}}_{{{{{\rm{p}}}}}}$ indicates the set of test proteins, ${M}_{{S}_{{{{{\rm{p}}}}}}}$ denotes the collection of generated molecules for the target protein ${S}_{{{{{\rm{p}}}}}}\in {{\mathbb{S}}}_{{{{{\rm{p}}}}}}$, *#* counts the number of molecules, and Set is an operator to remove the repeated molecules in the given set.
- **LogP**. A large LogP value indicates the substance is lipophilic, while a small LogP value means it is easy to dissolve in water. According to Ghose filter^33^, the LogP value of a druggable molecule should range from  −0.4 to  +5.6.
- **QED**. This score measures the drug-likeness and ranges from 0 to 1. A higher QED score indicates that a molecule is more likely to be a potential drug-like compound, with the desired molecular properties such as hydrogen bond acceptor, hydrogen bond donor, and polar molecular surface area^34^.
- **SA score**. The synthetic accessibility (SA) score indicates how difficult one molecule is to synthesize, which is calculated based on a combination of fragment contributions and a complexity penalty^35^. The range of the estimated SA metric is from 1 (easy to make) to 10 (very difficult to make).
- **NP-likeness**. Natural products play an important role in the history of drug discovery. Many drugs are natural products and their derivatives. The higher the score is, the more likely the molecule is to be a natural product. The calculated NP-likeness is typically in the range from -5 to 5^36^.

The reported results of “Known ligands”, SBMolGen, LiGANN, SBDD-3D, and BeamLmser are from AlphaDrug^15^. The “Known ligands” indicates the original molecules binding to protein targets in the database. The results of LiGANN^10^ were collected on the web-based application provided in the original paper. SBMolGen^37^ is developed from ChemTS^38^ for target-specific molecular generation. The results of SBDD-3D^18^ were based on the released codes and trained model published by the authors. BeamLmser applies the beam search on the pretrained Lmser Transformer^15^. The beam size of BeamLmser is set at 10 to collect 10 molecules for each test protein target. Besides the above representative baselines, we also test three recent advanced methods. The first is Pocket2Mol^20^, which uses the equivariant generative network and autoregressive sampling scheme to generate three-dimensional molecules. For Pocket2Mol, we utilize the official codes and trained model for sampling molecules. The second is TargetDiff^12^, which develops a three-dimensional equivariant diffusion model to sample molecules. For TargetDiff, we also use the officially released trained model and codes for sampling. We keep the sample numbers of Pocket2Mol and TargetDiff at 100 for each test protein, which is the default configuration to ensure the quality of generated molecules. To make a fair comparison with other methods, for each test protein target, we randomly select 10 molecules from the generated 100 molecules of Pocket2Mol and TargetDiff for the evaluation. The third is CProMG^28^, which proposes a multi-constraint autoregressive model to generate small molecules with controllable properties. We use the official codes and default configurations of CProMG to generate 10 molecules for each test protein with the pretrained CProMG-VQSLT model, which is trained to control multiple property metrics including the docking score, LogP, QED, and SA score that are evaluated here.

Besides the above basic generative models, there also emerges another kind of fundamental approach that integrates the powerful MCTS-based searching technique to better control the molecule generation procedure of the pretrained autoregressive generative models with the simulation feedback, and AlphaDrug and the proposed ParetoDrug fall into this kind. For AlphaDrug^15^ which utilizes MCTS with the pretrained Lmser Transformer model to generate molecules based on given protein targets, we run the official codes and set iteration times (IT) at 150 when selecting the next atom symbol in MCTS. For ParetoDrug which conducts Pareto MCTS with the same pretrained Lmser Transformer model, we also set IT at 150 and let it optimize all objectives (docking score, LogP, QED, SA score, and NP-likeness) synchronously except the unoptimizable Uniqueness, which is a statistic metric for all generated molecules. In addition, we set the metric value of LogP as 1 if the molecule’s LogP value is in the range of [ − 0.4, 5.6], and 0 otherwise. After each Pareto MCTS, ParetoDrug obtains a global pool of Pareto optimal molecules. We choose the molecule with the largest reward vector summation value from the pool, which means this molecule has top rankings in each property metric. When testing, we collect 10 generated molecules for each test protein target from AlphaDrug and ParetoDrug.

Additionally, we compare a multi-objective drug discovery algorithm REINVENT 4^39^ while its generation model is not conditioned on the protein information. It uses a reinforcement learning algorithm to generate optimized molecules compliant with a user-defined property profile defined as a multi-component score. We let REINVENT 4 optimize the docking score, LogP, QED, SA, and NP while setting their weights in the property profile all at 0.2. For each test protein target, we collected 10 molecules with the highest multi-component scores during the training process of REINVENT 4.

The results are shown in Table 1 and the direction of the arrow in the table means a better property score. The 95% confidence intervals for property scores of RL/MCTS are included. As we see, in terms of the docking score, ParetoDrug demonstrates superiority over all baselines except AlphaDrug. However, AlphaDrug is a single-objective target-aware drug discovery method that only optimizes the binding affinity. As AlphaDrug and ParetoDrug have the same iteration budgets (IT=150) for each atom symbol in sequence but ParetoDrug needs to optimize multiple objectives including the binding affinity, it is expected that ParetoDrug has a lower docking score than AlphaDrug. Meanwhile, although the docking score of ParetoDrug decreases slightly, other metrics including QED, SA score, and NP-likeness are improved significantly compared with AlphaDrug. Notably, QED changes from 0.4 to 0.6 (50% improvement) while NP-likeness changes from -0.9 to -0.4 (55.6% improvement). For the special LogP metric, although the average LogP value of AlphaDrug falls into the druggable molecule range, only 52.7% generated molecules of AlphaDrug satisfy the LogP range constraint if tested individually. On the contrary, 96.5% (83.1% improvement over AlphaDrug) generated molecules of ParetoDrug satisfy the LogP range constraint. These impressive results demonstrate that ParetoDrug is able to address the multi-objective target-aware drug discovery task by discovering novel compounds that possess multiple satisfactory properties including the binding affinity. On the other hand, we observe that the pretrained autoregressive Lmser Transformer with beam search (BeamLmser) cannot generate molecules with higher docking scores than the most recent TargetDiff. But with MCTS replacing beam search, AlphaDrug greatly boosts Lmser Transformer’s performance to find molecules with stronger binding affinity than BeamLmser even with the same docking time budgets^15^. Furthermore, ParetoDrug proposes the multi-objective Pareto MCTS to replace the MCTS used in AlphaDrug. With the same iteration times, ParetoDrug significantly improves multiple molecule properties compared with AlphaDrug while maintaining the docking score at the same level. Additionally, when compared with the multi-constraint conditional generation method CProMG, ParetoDrug has advantages in docking score, Uniqueness, SA score, and NP-likeness. In addition, the Uniqueness of CProMG is only 26.9% as it generates the same molecules for different protein targets, which is undesirable in de novo target-aware drug discovery tasks. Lastly, for REINVENT 4 which does not belong to the kind of target-aware drug discovery methods, we could see although it achieves superior performance in some metrics such as QED and NP, its docking score is much lower than ParetoDrug as it does not encode the protein-ligand prior to its generation model. This also indicates the importance of incorporating the protein target information into the molecule generation process as in the generative target-aware drug discovery methods.

**Table 1:** Average metric scores of generated molecules of each method (*n* = 1000 molecules) on the sampled 100 test proteins

Type	Methods	Docking score (*↑*)	Uniqueness (*↑*)	LogP	QED (*↑*)	SA (*↓*)	NP (*↑*)
Reference	Known ligands	9.8	–	2.2	0.5	3.3	–1.0
Generative model	LiGANN^10^	6.7	94.7%	2.9	0.6	3.0	–1.1
	SBMolGen^37^	7.7	100%	2.6	0.7	2.8	–1.2
	SBDD-3D^18^	7.7	99.3%	1.5	0.6	4.0	0.3
	Pocket2Mol^20^	8.1	99.8%	2.0	0.7	3.0	–0.2
	CProMG^28^	8.2	26.9%	1.9	0.8	2.9	–0.9
	BeamLmser^15^	8.5	98.1%	4.0	0.5	2.7	–1.0
	TargetDiff^12^	8.6	100%	2.9	0.5	5.2	0.6
RL/MCTS	REINVENT 4^39^	9.3 ± 0.1	98.7%	3.3 ± 0.0	0.8 ± 0.0	2.7 ± 0.0	0.7 ± 0.0
	AlphaDrug^15^	12.0 ± 0.1	99.9%	5.4 ± 0.1	0.4 ± 0.0	2.7 ± 0.0	-0.9 ± 0.0
	ParetoDrug	10.9 ± 0.1	99.9%	4.3 ± 0.1	0.6 ± 0.0	2.4 ± 0.0	-0.4 ± 0.0

The unit of docking score is kcal ⋅ mol^−1^.

The 95% confidence intervals for the property mean values of RL/MCTS methods are given. We conducted the two-sided *T* test to compare ParetoDrug and AlphaDrug, and the *p* values for Docking score, LogP, QED, SA, and NP are 3.4e–52, 1.1e–58, 8.0e–128, 3.6e–20, and 2.0e–62, respectively. We conducted the two-sided *T* test to compare ParetoDrug and REINVENT 4, and the *p* values for Docking score, LogP, QED, SA, and NP are 1.3e–110, 1.4e–104, 9.3e–265, 2.2e–21, and 8.5e–217, respectively.

**Fig. 1:**
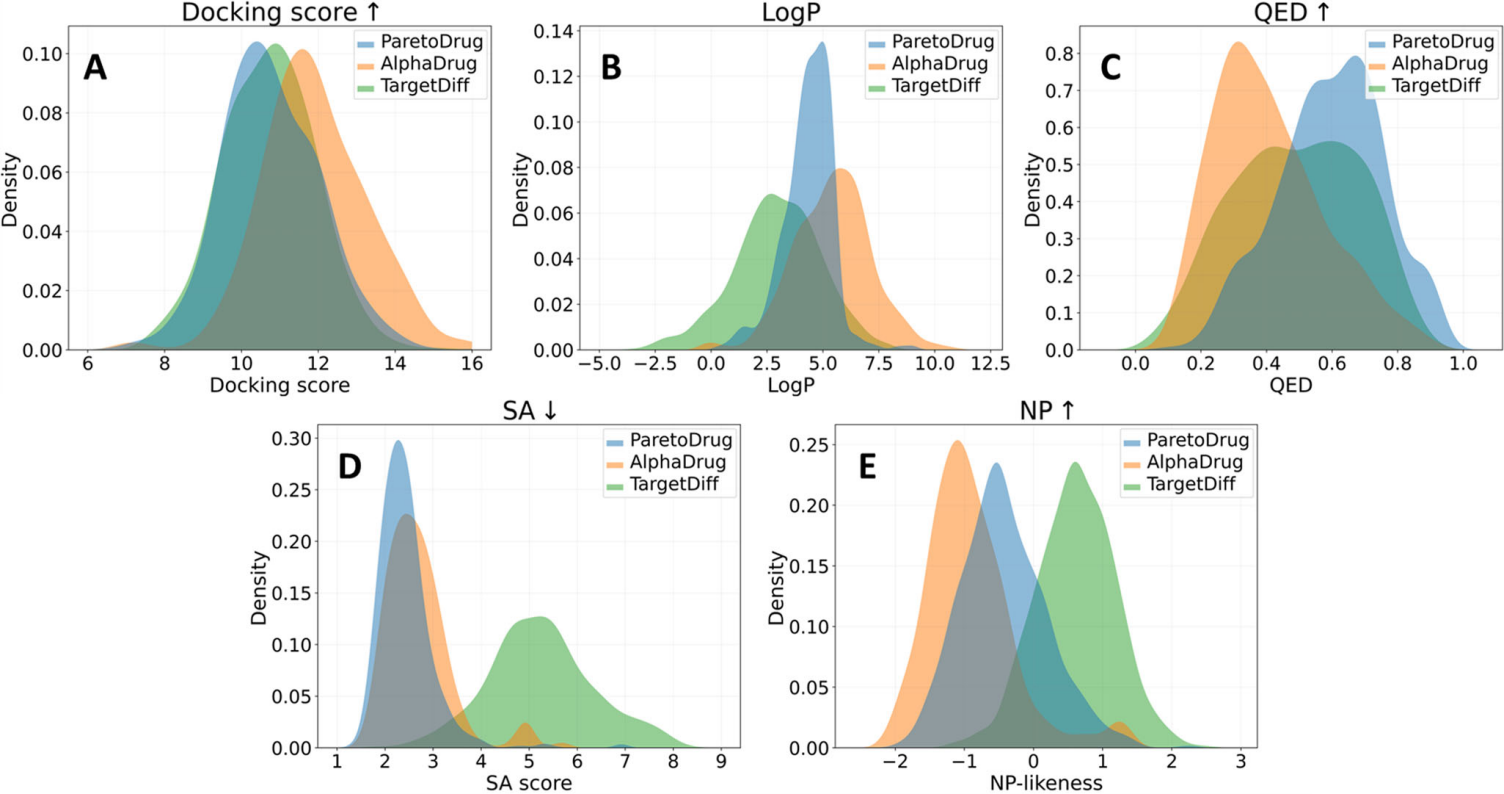
The molecular property distributions of generated molecules (*n* = 1000 molecules) by ParetoDrug, AlphaDrug, and TargetDiff respectively. **A** The docking score (kcal ⋅ mol^−1^) distributions of each method. **B** The LogP value distributions of each method. **C** The QED value distributions of each method. **D** The SA score distributions of each method. **E** The NP-likeness distributions of each method.

Next, we conduct the statistical analysis with kernel density estimate^40^, which is analogous to a histogram but endowed with benefits such as smoothness and continuity. The property distributions of molecules generated by TargetDiff, AlphaDrug, and ParetoDrug are shown in Fig. 1. For TargetDiff, here we use 10 molecules with the highest docking scores among the generated 100 molecules for each test protein to make an aligned comparison. We can see that although the docking score distributions of the three methods are similar while AlphaDrug is slightly better, other property distributions present differently. For LogP, ParetoDrug satisfies the range constraint of [ − 0.4, 5.6] while TargetDiff tends to generate more molecules with LogP values below the lower bound and AlphaDrug tends to generate more molecules with LogP values above the upper bound. Besides, ParetoDrug is able to generate more molecules with high QED values than the other two methods especially when QED is larger than 0.8 that molecules are very likely to be potential initiators of a drug candidate. Meanwhile, TargetDiff’s molecules are with significantly higher SA values than ParetoDrug, which means that TargetDiff’s molecules are much harder to synthesize. These statistical findings demonstrate that ParetoDrug has better molecule distributions than AlphaDrug and TargetDiff when taking multiple properties into account. More comparisons of computational efficacy, score distributions of a specific target, and the diversity of generated molecules between ParetoDrug and other methods could be referred to Supplementary Information C (and Supplementary Table 1), D (and Supplementary Fig. 1), and E (and Supplementary Table 2).

### Case studies for multi-objective target-aware drug discovery

Here we use two case studies of disease protein targets to show the molecule discovery ability of ParetoDrug for the multi-objective target-aware drug discovery tasks. The molecule objectives optimized by ParetoDrug are the docking score, LogP, QED, SA score, and NP-likeness. Additionally, the binding affinity is further validated by MM-GBSA^41,42^, which is a more accurate metric than docking scores but computationally expensive. For the analysis of the protein-ligand interactions, we use PLIP^43^ and detailed can be referred to Supplementary Information F.

#### Case 1: targeting FXR

**Fig. 2:**
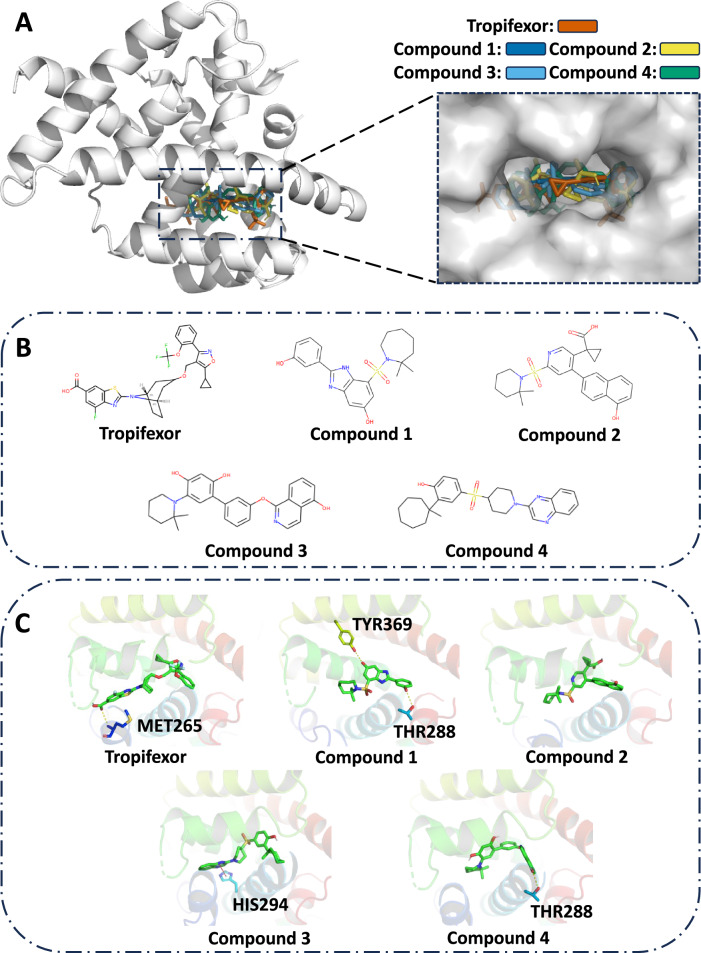
Static structural analysis of ligands binding to FXR (PDB ID: 7D42). Tropifexor is an agonist of FXR and Compounds 1–4 are found by ParetoDrug. Hydrogen bonds are displayed in yellow dashed lines and *π*-*π* interactions are in red. **A** Solvent-accessible surfaces of the binding pocket of FXR for Tropifexor and Compounds 1 to 4. **B** Chemical structures of Tropifexor and Compounds 1–4. **C** The binding poses of Tropifexor and Compounds 1–4.

Non-alcoholic fatty liver disease (NAFLD) is defined as the excessive and abnormal intracellular accumulation of lipids in the liver, primarily in the form of triglycerides^44,45^. Currently, NAFLD has been the most common cause of chronic liver disease, especially in Western countries, and the estimated prevalence of NAFLD is approximately 30% in the general population^46,47^. One of the best-known drugs for NAFLD is Tropifexor, which acts as an agonist of the farnesoid X receptor (FXR). The structural basis of Tropifexor as a potent and selective agonist of FXR is shown in Fig. 2A (PDB ID: 7D42)^48^. In this case study, we use ParetoDrug to discover potential drug molecules with desired computational properties for FXR. Using ParetoDrug, we collect 10 molecules and find four Pareto Dominate molecules compared with Tropifexor. The chemical structures of Tropifexor and the discovered ligands by ParetoDrug for FXR are shown in Fig. 2B. Table 2 shows the property metrics of different ligands. Our ParetoDrug model discovers multiple ligands that outperform Tropifexor on all the optimized properties. Especially, the SA scores of the new ligands are much lower than Tropifexor, which means that they are easier to synthesize. We also run AlphaDrug and TargetDiff to collect molecules for FXR, however, no Pareto Dominate molecule over Tropifexor is found for the two methods. For example, the best molecule from AlphaDrug (with the most number of better properties than Tropifexor) has the Docking score at 12.9, LogP at 4.1, QED at 0.3, SA at 2.6, and NP at -1.59. Compared with it, Compound 4 generated by ParetoDrug has 3 better properties (Docking score, QED, and NP) and 1 worse property (SA). Meanwhile, the best molecule from TargetDiff (with the most number of better properties than Tropifexor) has the Docking score at 12.8, LogP at 4.9, QED at 0.48, SA at 7.7, and NP at 1.01. Compared with it, Compound 4 generated by ParetoDrug has 3 better properties (Docking score, QED, and SA) and 1 worse property (NP). As shown in Fig. 2C, the docked poses and interactions of these four discovered compounds are quite different compared with Tropifexor. More specifically, one hydrogen bond forms between Tropifexor and the amino-acid residue MET265. At the same time, Compounds 1, 3, and 4 with new scaffolds form new hydrogen bonds with other residues (Compound 1 with THR288 and TYR369, Compound 3 with HIS294, and Compound 4 with THR288) while no hydrogen bond forms between Compound 2 and FXR.

**Table 2:** Metrics of generated molecules for protein target FXR

Ligands	Docking score (*↑*)	LogP	QED (*↑*)	SA (*↓*)	NP (*↑*)	MM-GBSA score (*↓*)
Tropifexor	12.3	7.3	0.21	4.6	–0.91	–57.80 ± 6.69
Compound 1	12.5	4.0	0.60	2.9	–0.37	–55.11 ± 5.61
Compound 2	12.3	5.4	0.51	2.8	–0.75	–60.30 ± 4.27
Compound 3	12.5	6.6	0.32	2.8	–0.04	–62.09 ± 4.72
Compound 4	13.0	4.7	0.55	3.1	–0.02	–64.82 ± 4.64

The unit of docking score and MM-GBSA score (*n* = 3 simulations for MM-GBSA) is kcal ⋅ mol^−1^.

The 95% confidence intervals of MM-GBSA scores are included. We conducted the two-sided *T* test to compare the MM-GBSA scores of each compound with Tropifexor. The *p* value is 0.5784 for Compound 1, 0.5713 for Compound 2, 0.3622 for Compound 3, and 0.1662 for Compound 4. These *p* values ( >0.05) indicate Compound 1–4 have the same level of binding free energies as the known drug Tropifexor.

Besides the docking score, MM-GBSA rescoring based on molecular dynamics simulations is used to further computationally validate the discovered compounds^41^. MM-GBSA uses molecular mechanics with generalized Born surface area to determine highly potential inhibitors for targets. The detailed settings of MM-GBSA are provided in Supplementary Information G. In this case, we use MM-GBSA to further validate the generated Pareto optimal molecules with promising docking scores. As shown in Table 2, the MM-GBSA scores indicate that the generated molecules have the same level of binding free energies as the known drug Tropifexor. Surprisingly, although two hydrogen bonds formed between Compound 1 and FXR, the MM-GBSA scores show no significant difference. Possibly because hydrogen bonding interaction is ignored in the MM-GBSA calculation.

#### Case 2: targeting PI3K-*γ*

**Fig. 3:**
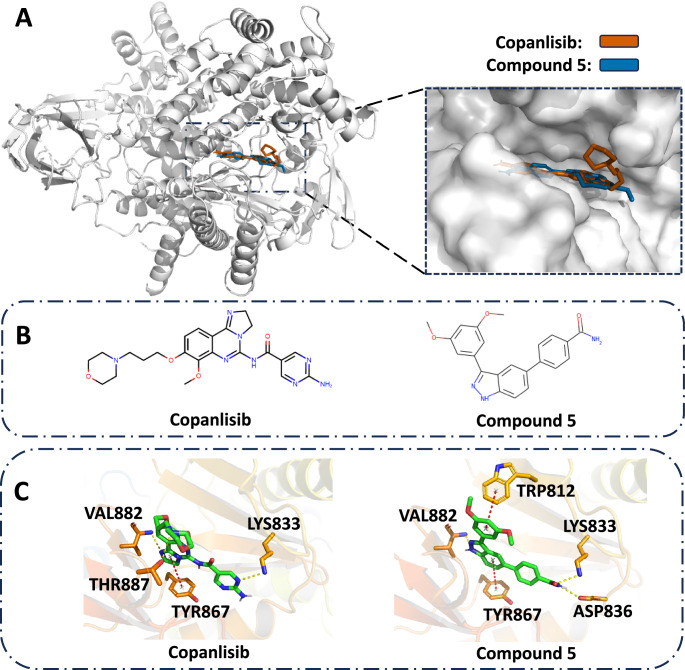
Static structural analysis of ligands binding to PI3K-*γ* (PDB ID: 5G2N). Copanlisib is a drug binding to PI3K-*γ* and Compound 5 is found by ParetoDrug. Hydrogen bonds are displayed in yellow dashed lines and *π*-*π* interactions are in red. **A** Solvent-accessible surfaces of the binding pocket of PI3K-*γ* for Copanlisib and Compound 5. **B** Chemical structures of Copanlisib and Compound 5. **C** The binding poses of Copanlisib and Compound 5.

Follicular lymphoma (FL) is a systemic neoplasm of the lymphoid tissue displaying germinal center B-cell differentiation, which belongs to a cancer that involves certain types of white blood cells known as lymphocytes. FL represents 5% of all hematological neoplasms and about 20-25% of all new non-Hodgkin lymphoma diagnoses in Western countries^49^. One of the best-known drugs for FL is Copanlisib, which has been shown to affect the survival and spread of cancerous B-cells. The structural basis of the PI3K-*γ* related to FL in complex with Copanlisib is shown in Fig. 3A (PDB ID: 5G2N)^50^. Here we use ParetoDrug to discover potential drug molecules with desired computational properties for PI3K-*γ*. We collect 10 molecules and find one Pareto Dominate molecule (Compound 5) compared with Copanlisib (Fig. 3B). As shown in Fig. 3C, Compound 5 found by ParetoDrug has three hydrogen bonds with surrounding residues (VAL882, ASP836, and LYS833) and two *π*-*π* stackings with surrounding residues (TRP812 and TYR867). Notably, the hydrogen bonds to VAL882 and LYS833 as well as *π*-*π* stacking to TYR867 also appear in Copanlisib’s docking interactions. Meanwhile, Table 3 shows the computational metric values of Copanlisib and Compound 5. Compound 5 is better than Copanlisib in terms of the optimized molecule metric objectives. However, the MM-GBSA scores indicate that the binding strength of Compound 5 decreases compared with Copanlisib (-46.48 kcal ⋅ mol^−1^ vs. -55.51 kcal ⋅ mol^−1^). The possible reason is that hydrogen bonding and *π*-*π* interactions are not considered in the energy terms of MM-GBSA. We also run AlphaDrug and TargetDiff to collect molecules for PI3K-*γ* and no Pareto Dominate molecule over Copanlisib is found by the two methods. For example, the best molecule from AlphaDrug (with the most number of better properties than Copanlisib) has the Docking score at 12.4, LogP at 4.35, QED at 0.41, SA at 5.0, and NP at 1.29. Meanwhile, the best molecule from TargetDiff (with the most number of better properties than Copanlisib) has the Docking score at 11.6, LogP at 3.2, QED at 0.56, SA at 3.9, and NP at 0.18. Although with some good properties, the two top molecules from AlphaDrug and TargetDiff cannot dominate the drug Copanlisib with worse SA.

**Table 3:** Metrics of the generated molecule for protein target PI3K-*γ*

Ligands	Docking score (*↑*)	LogP	QED (*↑*)	SA (*↓*)	NP (*↑*)	MM-GBSA score (*↑*)
Copanlisib	9.6	0.7	0.55	3.1	–1.06	–55.51 ± 1.75
Compound 5	10.3	4.0	0.55	2.2	–0.74	–46.48 ± 5.41

The unit of docking score and MM-GBSA score (*n* = 3 simulations for MM-GBSA) is kcal ⋅ mol^−1^.

The 95% confidence intervals of MM-GBSA scores are included. We conducted the two-sided *T* test to compare the MM-GBSA scores of Compound 5 with Copanlisib, and the *p* value is 0.0358.

While the in silico computational metrics of molecules discovered by ParetoDrug show promise in comparison to existing drugs, it is crucial to acknowledge that these molecules are still far from being drugs. Drug discovery is an extremely complicated process, and the current metrics for molecules cannot perfectly reflect the physicochemical properties required for a compound to be a drug. Nevertheless, we clearly see ParetoDrug’s promising potential in addressing multi-objective target-aware drug discovery tasks.

### Case study for multi-target drug discovery

Multi-target drug discovery can be considered a special case of multi-objective drug discovery where each protein target is going to be regarded as an objective to optimize. Until now, the study of multi-target target-aware drug discovery remains underexplored as it is challenging to consider the information of multiple protein targets at the same time to derive one ligand that could bind to all these given targets. Meanwhile, previous generative target-aware drug discovery works mainly focus on the single-target situation as there lack data sets to train the multi-target conditioned generative models. In this case study, we use ParetoDrug to perform a multi-target target-aware drug discovery task to design dual-functional inhibitors for both the HIV protease (HIV-PR) and HIV reverse transcriptase (HIV-RT). ParetoDrug is slightly modified to be compatible with this kind of task, and details are given in the Method section.

The crystal structures of HIV-PR and HIV-RT used here are 3A2O and 4G1Q^51^. Both structures are complexes with potent inhibitors solved at high resolution. We compare with LigBuilder V3^51^, the first de novo multi-target drug design program, and the variants of Pocket2Mol and TargetDiff extended by combining with screening. There are three different strategies in LigBuilder V3, including multi-target de novo design, multi-target growing, and multi-target linking. The best molecules for each strategy from the original paper are reported here. For the variants of Pocket2Mol and TargetDiff, we use each method to generate 100 molecules for each target. Then we use smina to screen the generated 200 molecules of each method to find the best molecule that has the best docking scores for both targets. We call the variants as Pocket2Mol-screen and TargetDiff-screen, and report the best molecules of each variant.

**Fig. 4:**
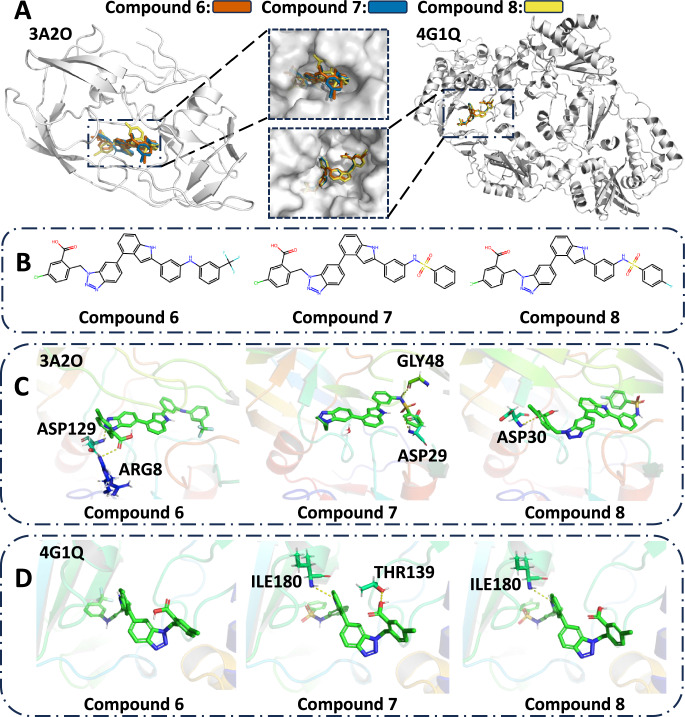
Static structural analysis of ligands binding to both the HIV-PR (PDB ID: 3A2O) and HIV-RT (PDB ID: 4G1Q). Compounds 6–8 are found by ParetoDrug. Hydrogen bonds are displayed in yellow dashed lines. **A** Solvent-accessible surfaces of the binding pockets of HIV-PR (left) and HIV-RT (right) for Compounds 6–8. **B** Chemical structures of Compounds 6–8. **C** The binding poses of Compounds 6 to 8 with HIV-PR. **D** The binding poses of Compounds 6–8 with HIV-RT.

Results of both the docking scores and MM-GBSA scores for each method’s best molecule are shown in Table 4. For ParetoDrug, we additionally report another two top molecules (Compound 7 and Compound 8) which are almost the same good as Compound 6 in terms of the docking scores. As shown in Table 4, the promising docking scores and MM-GBSA scores of Compounds 6–8 demonstrate that they are potential strong dual inhibitors to the given two protein targets in this task. Additionally, Fig. 4 shows Compounds 6–8 and their docking poses and interactions with HIV-PR (PDB ID: 3A2O) and HIV-RT (PDB ID: 4G1Q). Interestingly, the similar structures of Compounds 6 to 8 in Fig. 4B indicate that ParetoDrug found a chemical subspace of strong inhibitors for both the HIV-PR and HIV-RT targets.

**Table 4:** Docking and MM-GBSA scores of the generated molecules by baselines and ParetoDrug for protein targets HIV-PR and HIV-RT

Methods		Docking score (*↑*)		MM-GBSA score (*↓*)	
		HIV-PR	HIV-RT	HIV-PR	HIV-RT
LigBuilder V3	de novo	12.0	8.4	–30.38 ± 5.49	–47.82 ± 2.88
	growing	10.6	8.1	–25.18 ± 6.09	–38.95 ± 10.07
	linking	13.2	10.8	–38.04 ± 2.40	–59.36 ± 0.96
Pocket2Mol-screen		16.4	12.4	–35.03 ± 17.89	–60.87 ± 1.31
TargetDiff-screen		19.0	11.7	–44.59 ± 16.37	–66.36 ± 5.90
Compound 6 (ParetoDrug)		20.7	12.9	–67.78 ± 7.29	–78.51 ± 4.09
Compound 7 (ParetoDrug)		20.5	13.0	–61.37 ± 9.99	–86.30 ± 17.10
Compound 8 (ParetoDrug)		20.4	13.1	–53.64 ± 6.51	–77.23 ± 3.70

The unit of docking score and MM-GBSA score (*n* = 3 simulations for MM-GBSA) is kcal ⋅ mol^−1^.

The 95% confidence intervals of MM-GBSA scores are included. We conducted the *T* test with the alternative hypothesis that the MM-GBSA score on HIV-PR of Compound 6 is less than molecules from other baselines. All *p* values are less than 0.05. It is the same for HIV-RT.

### Case study for multi-target multi-objective drug discovery

**Fig. 5:**
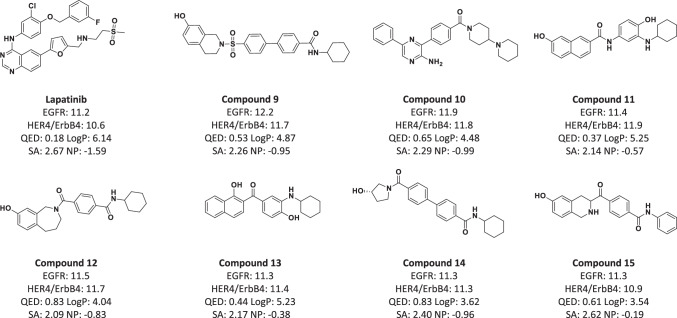
Property metric values of Lapatinib and its Pareto Dominate molecules (Compounds 9 to 15) found by ParetoDrug. EGFR means the ligand’s docking score to EGFR (PDB ID: 1XKK) while HER4/ErbB4 means the ligand’s docking score to HER4/ErbB4 kinase (PDB ID: 3BBT), and the unit for the two docking scores is kcal ⋅ mol^−1^.

We have shown ParetoDrug’s promising ability for both the multi-objective target-aware drug discovery and multi-target drug discovery tasks separately. Naturally, a more attractive and challenging task is the multi-target multi-objective drug discovery task where the generated molecules need to bind to a given set of protein targets while manifesting other desired computational molecule properties. To the best of our knowledge, there are no published works yet to specifically address this kind of task. Here we use Lapatinib as a case study to evaluate ParetoDrug’s ability for the multi-target multi-objective drug discovery task. Lapatinib is a dual tyrosine kinase inhibitor that interrupts the EGFR pathway and inhibits HER4/ErbB4 Kinase for the treatment of breast cancer^52,53^. 1XKK and 3BBT are respectively the PDB IDs of crystal structures for Lapatinib binding to EGFR^54^ and the HER4/ErbB4 kinase^55^. In this task, we configure ParetoDrug to bind to both protein targets while optimizing LogP, QED, SA, and NP metrics synchronously with IT at 150. We compare the generated molecules in the global Pareto pool with the known dual-inhibitor drug Lapatinib. Impressively, plenty of Pareto Dominate molecules over Lapatinib are discovered by ParetoDrug, as shown in Fig. 5. Furthermore, the QED values of Compound 12 and Compound 14 are greater than 0.8, which indicates the two molecules are potential initiators for a drug.

## Discussion

In this work, we divide multi-objective drug discovery methods into three kinds based on whether they use the target protein information. The representative works of each kind of method and their comparisons are provided in Table 5. The first kind is the ligand-based method such as MolGPT^22^, which utilizes the conditional transformer to generate molecules that satisfy multiple inputting property constraints. However, the ligand-based method does not consider protein information and thus cannot guarantee to generate molecules with high binding affinity to a given protein target.

**Table 5:** Comparison of different multi-objective drug discovery methods

Method	Target-based type	Multi-objective type	Objectives
MolGPT^22^	Ligand-based	constraint	QED, SA, LogP, TPSA^72^
MolSearch^24^	Target-scoring-based	optimization	QED, SA, docking^a^ (GSK3*β*, JNK3)
MCMG^21^	Target-scoring-based	constraint & optimization	QED, SA, docking^b^ (GSK3*β*, JNK3, DRD2)
RationaleRL^23^	Target-scoring-based	constraint & optimization	QED, SA, docking^c^ (GSK3*β*, JNK3)
GENERA^25^	Target-scoring-based	optimization	docking^d^ (ACE2)
REINVENT 4^39^	Target-scoring-based	optimization	User-defined objectives including docking^e^
CProMG^28^	Target-aware	constraint	QED, SA, LogP, TPSA, docking^f^
ParetoDrug	Target-aware	optimization	QED, SA, LogP, NP-likeness, docking^g^

^a^Docking scores of GSK3*β* and JNK3 are predicted by pretrained random forest models^23^.

^b^Docking scores of GSK3*β* and JNK3 are predicted by pretrained random forest models^23^. The docking score of DRD2 is predicted by a support vector machine classifier with a Gaussian kernel^73^.

^c^Docking scores of GSK3*β* and JNK3 are predicted by pretrained random forest models^23^.

^d^Docking scores of ACE2 are predicted by PLANTS^74^ and Glide^75^.

^e^In this study, we configure the optimization objectives of REINVENT 4 as QED, SA, LogP, NP-likeness, and Docking score. The docking score of a given protein target (not limited to specific targets) is predicted by smina^32^.

^f^Docking score of a given protein target (not limited to specific targets) is predicted by Autodock Vina^76^.

^g^Docking score of a given protein target (not limited to specific targets) is predicted by smina^32^.

The second kind is the target-scoring-based method that employs a docking scoring function to predict the binding affinity of the generated molecules to the given protein target. In this way, although the target-scoring-based method also does not explicitly consider the target protein information, the binding affinity scores could be optimized by optimization techniques such as reinforcement learning and genetic algorithm. For example, Wang et al., propose MCMG^21^ to combine conditional transformer, knowledge distillation, and reinforcement learning to generate molecules that satisfy multiple constraints including binding to targets such as GSK3*β* and JNK3. However, MCMG does not incorporate the target protein information into the generation process of its model and needs to design a reward that is a linear combination of each metric. Recently, MolSearch^24^ is proposed to use multi-objective MCTS to generate molecules based on molecule fragments. However, MolSearch is a pure search-based method with predefined massive rules to modify molecules and also does not consider the target protein information. Furthermore, REINVENT 4^39^ uses a reinforcement learning algorithm to generate optimized molecules compliant with a user-defined property profile defined as a multi-component score. However, it is not a generative target-aware method although it optimizes multiple objectives while treating the binding affinity as a standard optimizing objective. The lack of specific protein information in these target-scoring-based methods makes the optimization of binding affinity objective inefficient and the trained models cannot be generalized to other target proteins. Therefore, this kind of method is different from the mainstream target-aware molecule generation in that the protein-ligand interactions are modeled in the molecule generation process. Meanwhile, most of them are only evaluated on several case studies which limits the assessment of their generality on various target proteins. For example, RationaleRL^23^, MCMG^21^, and MolSearch^24^ optimize the molecule’s binding affinity to GSK3*β* and JNK3, which is predicted by random forest models pretrained on the data sets^56^ that contain samples of positive and negative compounds to the GSK3*β* and JNK3 targets, and are not available for most protein targets.

The third kind of method is the multi-objective target-aware molecule generation, which models the protein-ligand interactions to generate the molecules with high binding affinity to the inputting protein target. Recently, CProMG^28^ is proposed to use the conditional multi-constraint autoregressive framework to generate molecules owning desired property constraints in a controllable manner. However, the ability of CProMG largely depends on the quality of data used to train the model and it does not involve an optimization process for a comprehensive searching in the chemical space. Compared with CProMG, ParetoDrug does not use a multi-constraint generative model. Instead, ParetoDrug employs the Pareto MCTS to optimize multiple objectives synchronously by searching desired molecules with the guidance of the pretrained autoregressive molecule generative model. Also as shown in the benchmark experiment, ParetoDrug achieves better multi-objective metrics of the generated molecules when compared with CProMG on all the property objectives except QED.

In conclusion, in this work, we propose ParetoDrug to fulfill the gap of multi-objective target-aware drug discovery in the field of deep learning-based drug discovery. ParetoDrug is an autoregressive Pareto MCTS algorithm that integrates the pretrained autoregressive generative model to search desired multi-objective molecules in an atom-by-atom way with the help of Pareto MCTS. We perform the evaluation of ParetoDrug on a standard benchmark setting with various baselines. The benchmark results show that ParetoDrug achieves multiple satisfactory molecule properties including binding affinity while previous single-objective methods cannot. We further conduct the case studies of the multi-objective target-aware drug discovery tasks for two known drugs, the multi-target drug discovery task for HIV-related disease targets, and the multi-target multi-objective drug discovery task for a dual inhibitor. In these case studies, new molecules discovered by ParetoDrug exhibit high potentials that Pareto Dominate the known drugs of the disease targets on all required property objectives. In conclusion, ParetoDrug demonstrates its ability to handle the challenging multi-objective target-aware drug discovery tasks and its superiority in searching in the large and complex chemical space for novel compounds that possess multiple promising properties including binding affinity.

For future work, on the one hand, making ParetoDrug compatible with more recent advanced autoregressive molecule generative models such as the Diffusion model is highly promising. On the other hand, extending ParetoDrug into the multi-objective design of protein, polypeptide, and nucleic acid drugs also holds significant potential.

## Methods

In this section, we first formulate the target-aware drug discovery task as a Markov decision process. Then we introduce the concepts of Pareto Dominate and Pareto Front in the multi-objective optimization domain. Finally, we propose the framework of ParetoDrug designed for the multi-objective target-aware drug discovery task and the multi-target target-aware drug discovery task.

### Problem definition

Target-aware molecule generation can be formulated as a Markov decision process (MDP)^57^ given that the next atom to be chosen only depends on the generated molecule fragment and the protein target. The MDP can be defined as *M* = (*S*, *A*, *P*, *R*) where *S* denotes the set of states that describe the current molecule fragment and the protein, *A* denotes the set of actions that indicate the chosen atom symbol to be added to the current molecule fragment, and *P*: *S* × *A* → *S* is the state transition function where the molecule fragment incorporates the chosen atom symbol to grow up to a new molecule fragment. $R:S\to {{\mathbb{R}}}^{d}$ is the reward function based on the current state. In target-aware molecule generation, the reward to evaluate the generated molecule is usually available at the terminal state, which is a typical sparse-reward setting. If *d* > 1, multiple reward objectives are considered such as strong binding affinity, high drug-likeness, and low toxicity in drug discovery. The goal is to take the action that maximizes the expected episodic reward $\overline{R}(s,a)$, which can be approximated under repeated rollouts^58^ as

2 $$\overline{R}(s,a)=\frac{1}{N(s,a)}\sum\limits_{j=1}^{N(s)}{{\mathbb{I}}}_{j}(s,a){r}^{j}(s),$$

where *N*(*s*) denotes the rollout times starting from state *s* and *N*(*s*, *a*) is the times that action *a* has been taken from state *s*. ${{\mathbb{I}}}_{j}(s,a)$ is an indicator function with value 1 if action *a* is selected from state *s* at the *j*th rollout round, 0 otherwise. *r* ^*j*^(*s*) is the final reward to evaluate the final generated molecule at the terminal state for the *j*th rollout round starting from state *s*. A larger $\overline{R}(s,a)$ value indicates a higher expected reward by taking action *a* from state *s*.

### Multi-objective optimization

Multi-objective optimization (also known as Pareto optimization) is concerned with optimization problems involving more than one objective function to be optimized simultaneously^59^, which has been applied in many fields. In multi-objective optimization, there does not typically exist a feasible solution that maximizes all objective functions at the same time. Therefore, attention is paid to Pareto optimal solutions^60^, which cannot be improved in any of the objectives without degrading at least one of the other objectives. In mathematical terms, a feasible vector ${{{{\bf{X}}}}}\in {{\mathbb{R}}}^{d}$ is said to Pareto Dominate another vector ${{{{{\bf{X}}}}}}^{{\prime} }\in {{\mathbb{R}}}^{d}$ is defined as below^61^.

#### Definition 1

Pareto Dominate. Given two vectors **X** = (*x*_1_, …, *x*_*d*_) and ${{{{{\bf{X}}}}}}^{{\prime} }=({x}_{1}^{{\prime} },\ldots ,{x}_{d}^{{\prime} })$, **X** is said to dominate ${{{{{\bf{X}}}}}}^{{\prime} }$, i.e., ${{{{\bf{X}}}}}\succcurlyeq {{{{{\bf{X}}}}}}^{{\prime} }$ if and only if ${x}_{i}\ge {x}_{i}^{{\prime} },\forall i=1,\ldots ,d$. **X** is said to strictly dominate ${{{{{\bf{X}}}}}}^{{\prime} }$, i.e., ${{{{\bf{X}}}}}\succ {{{{{\bf{X}}}}}}^{{\prime} }$ if and only if ${{{{\bf{X}}}}}\succcurlyeq {{{{{\bf{X}}}}}}^{{\prime} }$ and  ∃ *i* such that ${x}_{i} > {x}_{i}^{{\prime} }$.

A vector ${{{{{\bf{X}}}}}}^{* }\in {{\mathbb{R}}}^{d}$ is called Pareto optimal if there does not exist another vector that Pareto Dominates it. The set of Pareto optimal vectors ${{\mathbb{X}}}^{* }$, called Pareto Front, is defined as below.

#### Definition 2

Pareto Front. Given a set of vectors ${\mathbb{X}}\subset {{\mathbb{R}}}^{d}$, the non-dominant set ${{\mathbb{X}}}^{* }\in {\mathbb{X}}$ is defined as ${{\mathbb{X}}}^{* }=\{{{{{\bf{X}}}}}\in {\mathbb{X}}:\nexists {{{{{\bf{X}}}}}}^{{\prime} }\in {\mathbb{X}}\,s.t.\,{{{{{\bf{X}}}}}}^{{\prime} }\succ {{{{\bf{X}}}}}\}$.

In drug molecule design, the optimization or constraint of multiple properties is a pervasive requirement. For instance, for a new drug to be successful, it must simultaneously be potent, bioavailable, safe, and synthesizable, with these properties being often competing^62^. As Pareto optimization is capable of discovering a set of solutions that reveal trade-offs among objectives and relies on no prior measure of the importance of competing objectives, it is believed as the most robust approach to multi-objective drug discovery^62^. Next, we introduce how to utilize the concepts of Pareto Dominate and Pareto Front to construct the ParetoDrug framework for multi-objective target-aware drug discovery with the help of MCTS and the pretrained autoregressive generative model.

### ParetoDrug

**Fig. 6:**
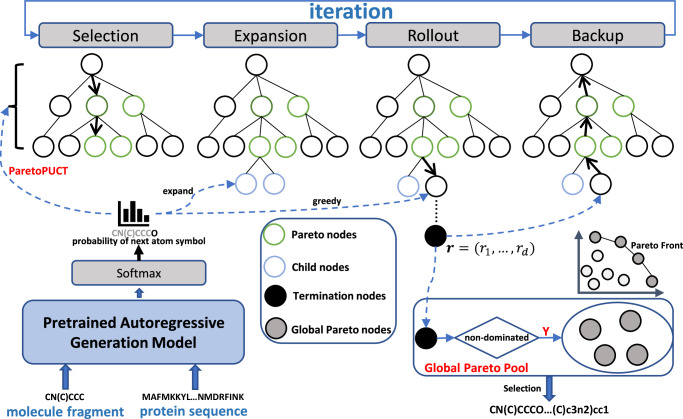
The framework of ParetoDrug. The pretrained autoregressive generative model gives the probability distribution of the next atom symbol. The ParetoPUCT balances the exploration and exploitation when searching for the next atom symbol in the Selection step. ParetoDrug maintains a global pool of Pareto optimal molecules, which are updated during Pareto MCTS.

To solve the challenging multi-objective target-aware drug discovery task, which requires generating molecules with multiple desired properties including the strong binding affinity to specified protein targets, we propose an autoregressive Pareto MCTS generation algorithm called ParetoDrug. First, ParetoDrug employs existing pretrained autoregressive generative models to provide exploration guidance toward desired molecules during searching. Based on both the protein context and intermediate molecule fragment, the pretrained autoregressive model predicts the probability of the next atom symbol to be added to the current molecule fragment. Second, with exploration guidance from the pretrained autoregressive model, ParetoDrug performs Pareto MCTS to progressively find Pareto optimal molecules with multiple desired properties. Third, to achieve the exploration-exploitation balance during searching, we propose the ParetoPUCT selection criterion to determine the next atom symbol in the Selection step of Pareto MCTS. Through these three key components, ParetoDrug is able to generate high-quality molecules for multi-objective target-aware drug discovery tasks. The overall framework of ParetoDrug is shown in Fig. 6. Next, we explain the details including Pareto MCTS, ParetoPUCT, and how to extend ParetoDrug into the case of multi-target target-aware molecule generation.

### Pareto MCTS

In MCTS, pretrained neural networks based on the expert data could be used for the guidance of action selection^63^ and this idea has been extended into single-objective target-aware drug discovery^15^. Similarly, to enable the exploration guidance to desired molecules with strong binding affinity to specified protein targets, ParetoDrug employs an existing pretrained autoregressive generative model^15^ to predict the next atom symbol given the protein target and current molecule fragment. The protein target is represented by the amino acid sequence. The molecule fragment is based on SMILES^64^, which describes molecules with short ASCII strings. The autoregressive generative model includes a protein encoder based on the protein’s amino acid sequence and a ligand molecule decoder. At each step, the protein encoder receives the target protein sequence and outputs the protein embedding into the ligand decoder. Next, the ligand decoder predicts the probability of the next atom symbol based on both the protein embedding and intermediate molecule fragment from the last step. This autoregressive generative model is pretrained on the protein-ligand data set and used in MCTS.

When generating molecules with the pretrained autoregressive generative model, although we could obtain the ligand molecule in a greedy manner by taking the next atom symbol with the maximum probability, it is prone to be stuck in a local optimum due to the unpredictable complexity of the chemical space. At the same time, the predicted atom with the maximum probability does not mean that it must be in the optimal molecule that satisfies multiple required properties as the pretrained model is not optimized for these properties. To address the above difficulties for the multi-objective target-aware drug discovery, we propose ParetoDrug, which employs the Pareto MCTS to enable a synchronous optimization of multiple properties together with the help of a pretrained autoregressive model for selection guidance. Next, we introduce Pareto MCTS.

Pareto MCTS^65^ extends basic MCTS^66^ to optimize multiple objectives, which adopts a tree structure to perform simulation iterations and estimates action values to guide searching. The Pareto MCTS procedure consists of four steps per iteration:

- **Selection**. Each iteration starts from the current root node *a*_*τ*_ and the best child is recursively selected until a leaf node *a*_*τ*+*l*_ after *l* selections, i.e., a node that has not been expanded or terminated, is reached. For each selection *t* ∈ [1, *l*], we need a selection criterion to determine which child node is the best to be chosen. This criterion balances between exploitation and exploration to avoid being trapped in local optimums and is given in Eq. (6).
- **Expansion**. Given a selected leaf node *a*_*τ*+*l*_, the probability *P*(*a*∣*C*_*τ*+*l*_) for each expandable atom symbol *a* ∈ *A* is computed by the pretrained autoregressive generative model. *C*_*τ*+*l*_ = {*S*_p_, *m*_*τ*+*l*_} is the state context with the target protein sequence *S*_p_ and the current simulated intermediate molecule fragment *m*_*τ*+*l*_ = *a*_1_ ⋯ *a*_*τ*_*a*_*τ*+1_ ⋯ *a*_*τ*+*l*_. Here *A* is the legal action space, i.e. the SMILES vocabulary of molecules, under the given state context. The expanded child nodes of *a*_*τ*+*l*_ are added to the tree and initialized immediately.
- **Rollout**. The value of the reached leaf node *a*_*τ*+*l*_ is evaluated by a fast rollout. From the leaf node, MCTS recursively generates the next state until termination and receives the reward of the final molecule at the termination state. During the rollout, each atom symbol is selected in a greedy manner according to the predicted probability given by the pretrained autoregressive network until a terminal symbol *a*_*τ*+*L*_ is generated or the tree reaches a maximum depth. The path from the initial atom symbol to the terminal atom symbol forms a complete molecule *m* = *a*_1_ ⋯ *a*_*τ*_*a*_*τ*+1_ ⋯ *a*_*τ*+*L*_. The reward **r** of the final molecule *m* is then evaluated based on the molecule property metrics. Specifically, the binding affinity is computed by the docking function *f*(*S*_p_, *m*) such as smina^32^. The reward **r** is calculated as defined in Eq. (3) by normalizing the property metric in each dimension.
- **Backup**. The reward is backpropagated along the visited nodes to update their statistics until the root node. The detailed updating process for tree nodes is elaborated in Eq. (4) with the defined reward vector **r** for nodes.

When performing Pareto MCTS, we maintain a global pool of all the Pareto optimal molecules found so far to represent the molecule Pareto Front as defined by Definition 2. We update the global pool of Pareto optimal molecules by adding newly generated Pareto optimal molecules and removing invalid ones if they are Pareto Dominated by the global pool’s new coming molecules. The molecule comparison is based on the reward vector **r** defined as follows. For each generated molecule in the rollout with the property metric vector ${{{{\bf{h}}}}}=({h}_{1},\ldots ,{h}_{d})\in {{\mathbb{R}}}^{d}$, the reward vector ${{{{\bf{r}}}}}=({r}_{1},\ldots ,{r}_{d})\in {{\mathbb{R}}}^{d}$ of this molecule is defined as

3 $${r}_{i}=\frac{1}{{N}_{{{{{\rm{P}}}}}}}\sum\limits_{k=1}^{{N}_{{{{{\rm{P}}}}}}}{\mathbb{I}}[{h}_{i}\ge {h}_{i}^{k}],\forall i=1,\ldots ,d,$$

where *N*_P_ is the number of Pareto optimal molecules in the global pool, ${h}_{i}^{k}$ is the *i*th property metric of the *k*th Pareto optimal molecule, and *h*_*i*_ is the *i*th property metric of the current generated molecule to be compared. ${\mathbb{I}}$ is the indicator function with value 1 if the condition ${h}_{i} > {h}_{i}^{k}$ is satisfied, 0 otherwise. The calculation of reward **r** treats each dimension separately, regardless of their scale difference, which gains an advantage over methods that aggregate all dimensions into one score using predefined weights^24^. With the reward vector, the Backup step is performed as

4 $${N}_{a}\leftarrow {N}_{a}+1,{{{{{\bf{W}}}}}}_{a}\leftarrow {{{{{\bf{W}}}}}}_{a}+{{{{\bf{r}}}}},a\leftarrow \,{\mbox{parent of}}\,\,a,$$

where *N*_*a*_ is the total times that node *a* has been selected and **W**_*a*_ is the cumulative reward vector of node *a*. For each selection *t* ∈ [1, *l*], the statistics of node *a*_*τ*+*t*_ are updated by adding the reward vector of the node *a*_*τ*+*l*_’s rollout result to **W**_*a*_ and increasing the visiting times *N*_*a*_ by 1.

### ParetoPUCT

The most important step of MCTS is the Selection step where a criterion is needed to select the next child node by comparing all child nodes. The most commonly used criterion is the upper confidence bound^67^ in which a child node is selected to maximize

5 $$U=\frac{{W}_{a}}{{N}_{a}}+\sqrt{\frac{2\ln N}{{N}_{a}}},$$

where *N* is the total times of iterations and *N*_*a*_ is the times of node *a* being selected. *U* is a scalar used to select the best child node with the largest value. However, in the multi-objective target-aware drug discovery, the reward becomes a vector and *U* is not applicable for the comparison of vectors. At the same time, we also want to utilize the pretrained autoregressive generative model to provide the exploration guidance in the chemical space^15,68^ when selecting the next child node in the Selection step.

Therefore, here we propose ParetoPUCT that extends the scalar predictor upper confidence bound applied to trees (PUCT) selection criterion^69^ with the concepts of Pareto Dominate and Pareto Front into a vectorial selection criterion for the multi-objective MCTS^65^. At each selection *t*, we first compute a selection score vector for each candidate child node as

6 $${{{{{\bf{U}}}}}}_{{{{{\rm{p}}}}}}({C}_{\tau +t-1},a)=\frac{{{{{{\bf{W}}}}}}_{a}}{{N}_{a}}+cP(a| {C}_{\tau +t-1})\frac{\sqrt{N}}{1+{N}_{a}},$$

where *c* is a constant that controls the degree of exploration. Here **W**_*a*_ is the cumulative reward vector for node *a*. The $\frac{\sqrt{N}}{1+{N}_{a}}$ part guides MCTS to initially prefer to visit the nodes with a low number of visits. At the same time, the *P*(*a*∣*C*_*τ*+*t*−1_) part tends to visit the atom nodes that are probably to produce a molecule with strong binding affinity to the protein target indicated by the pretrained autoregressive generative model. Furthermore, the $\frac{{{{{{\bf{W}}}}}}_{a}}{{N}_{a}}$ makes ParetoDrug exploit the nodes with multiple high property metrics while *c* balances the exploitation and exploration. As the **U**_p_ score is in the vector from for each child node *a*, to determine which child node to be selected, ParetoPUCT constructs a Pareto Front for those child nodes that are not Pareto Dominated by other child nodes by comparing their **U**_p_ score vectors. Each child node in the resulting Pareto Front cannot be replaced by a better child node and thus becomes the candidate node for the selection. Finally, ParetoPUCT selects a node from the Pareto Front of the candidate child nodes uniformly at random.

### Modifications of ParetoDrug for multi-target target-aware molecule generation

As the multi-target target-aware molecule generation involves multiple protein targets, we modify the ParetoPUCT node selection criterion to handle multiple predictions from the pretrained autoregressive generative model for different protein targets. Therefore, we propose the Multi-Target ParetoPUCT (M-ParetoPUCT) defined as

7 $${{{{{\bf{U}}}}}}_{{{{{\rm{mp}}}}}}=\frac{{{{{{\bf{W}}}}}}_{a}}{{N}_{a}}+cf({P}_{1}(a| {C}_{1,\tau +t-1}),\ldots ,{P}_{m}(a| {C}_{m,\tau +t-1}))\frac{\sqrt{N}}{1+{N}_{a}},$$

where there are *m* predictions for the next node *a* and *c* is a constant to control the exploration degree. For the prediction fusing function *f*(*P*_1_(*a*∣*C*_1,*τ*+*t*−1_), …, *P*_*m*_(*a*∣*C*_*m*,*τ*+*t*−1_)), each prediction is a distribution of the next atom symbol with the inputting of the molecule’s SMILES string representation^64^ and specified target protein’s amino acid sequence. Here *P*_*i*_(*a*∣*C*_*i*,*τ*+*t*−1_) is the neural network prediction for the *i*th target pretrained on the protein-ligand data set. As the distributions are on the same action set, we use the mean-pooling operation for *f* as

8 $$f({P}_{1}(a| {C}_{1,\tau +t-1}),\ldots ,{P}_{m}(a| {C}_{m,\tau +t-1}))=\frac{\mathop{\sum }_{i = 1}^{m}{P}_{i}(a| {C}_{i,\tau +t-1})}{m},\forall a\in A.$$

This mean-pooling operation keeps the probabilities of the possible next atom symbols for each protein target. Meanwhile, it enhances the probabilities of the next atom symbol if it is predicted to be preferred by all the given protein targets.

After a leaf node *a*_*τ*+*l*_ is selected, we need to expand it. The probability for each expandable atom symbol is computed the same as Eq. (8) from multiple predictions of the pretrained autoregressive model on multiple protein targets. Each child node *a* of *a*_*τ*+*l*_ is initialized to $\{{N}_{a}=0,{{{{{\bf{W}}}}}}_{a}={{{{\bf{0}}}}},\frac{\mathop{\sum }_{i = 1}^{m}{P}_{i}(a| {C}_{i,\tau +t-1})}{m}\}$.

### Statistics and reproducibility

Data manipulation and processing analyses were conducted using the packages Python (version 3.7), Biopython (version 1.79), Pandas (version 1.3.4), MMseqs2 (version 13.45111), RDKit (version 2020.09.5), PyTorch (version 1.13.1), and Openbabel (version 3.1.1). We used PyMOL (version 2.6.0a0) to analyze the protein structures. We used AMBER22 package to calculate the MM-GBSA scores (Supplementary Information G). We used PLIP 2021 to analyze protein-ligand Interactions Supplementary Information F. We use smina (version 2020.12.10) to calculate the docking score. The molecule property distributions are drawn by Matplotlib (version 3.4.3) and Seaborn (version 0.12.2), where the function “kdeplot” is called for kernel density estimate. The *T* test and *p* value calculation in this article are conducted with SciPy (version 1.9.1).

### Supplementary information

Supplementary Information

## Data Availability

The training and testing data of the autoregressive generative model used in ParetoDrug is processed from BindingDB and PDBbind, and is the same as in AlphaDrug and could be obtained from https://github.com/CMACH508/AlphaDrug. For the multi-objective target-aware drug discovery and multi-target drug discovery case studies, the PDB and ligand files of 7D42, 5G2N, 3A2O, 4G1Q, 1XKK, and 3BBT are downloaded from RCSB Protein Data Bank.

## Code availability

The source code of this study is publicly available from the GitHub repository: https://github.com/CNDOTA/ParetoDrug. We also provide the Google Colab version of ParetoDrug, which could be directly run online. The numerical source data for graphs and charts in this article is provided in Figshare with DOI^70^. We also deposit the data and codes of ParetoDrug in Figshare with DOI^71^.
