# Supplementary material for: Enabling target-aware molecule generation to follow multi objectives with Pareto MCTS
Source: Commun Biol. 2024 Sep 2;7:1074. doi: 10.1038/s42003-024-06746-w (PMC11368924; doi:10.1038/s42003-024-06746-w)
Supplement: Supplementary file 1 — Supplementary Information [file 42003_2024_6746_MOESM1_ESM.pdf]

Enabling Target-Aware Molecule Generation to Follow Multi Objectives with Pareto MCTS Supplementary Information

A. Experimental Setup

We follow Qian et al.[1], who use the public database of protein-ligand pairs BindingDB[2] and filter the crude database with criteria similar to Grechishnikova[3]. The data set for the pretrained autoregressive generative model contains 192,712 protein-ligand pairs for training and 17,049 pairs for validation[1].

The pretrained autoregressive generative model is based on the Lmser Transformer[1], which is able to generate molecules with strong binding affinity to target proteins. We use the released pretrained model weights provided in AlphaDrug and include the model in the code repository. For the hardware to run ParetoDrug, as it only involves the inference part of the pretrained model, an NVIDIA GPU with more than 2GB memory and 8 cores is enough to run a single instance. The code of ParetoDrug is based on Python and PyTorch and our experiments are conducted on Ubuntu 22.04.2 LTS. For the docking program smina[4], we install it with the Anaconda toolkit. To compute the docking score $f(S_p, m)$, smina needs the Protein Data Bank (PDB) file of the corresponding protein sequence S_p and the molecule file of the corresponding molecule SMILES of m. The PDB files could be downloaded from the PDBbind website and are also provided in the code repository. The molecule file could be converted from the molecule SMILES by RDKit. The other running dependencies are described in the code repository and we provide the command lines to install these dependencies.

For the case studies, the PDB and ligand files of 7D42, 5G2N, 3A2O, 4G1Q, 1XKK, and 3BBT are downloaded from RCSB Protein Data Bank[5]. The PDB files are further cleaned to remove ligand atoms. The reference docking ligands in the protein-ligand complexes 7D42, 5G2N, 1XKK, and 3BBT are also the known drugs.

B. Hyperparameter Setup

For the hyperparameter settings of Pocket2Mol, TargetDiff, and CProMG, we follow the official configurations of each method and uses pretrained model weights released by their authors. For AlphaDrug, we follow the official configuration and only change

the iteration time from 50 to 150 to ensure the same computational budgets as ParetoDrug. The hyperparameters of ParetoDrug are available in the code repository. The iteration time of the Pareto MCTS part in ParetoDrug is also set at 150.

C. Comparison of Computational Efficacy

We compare the computational efficacy of the RL or MCTS-based methods including AlphaDrug, REINVENT 4, and ParetoDrug with the same hard computational resources including 1 NVIDIA GPU and 8 CPU cores. We use the first protein target (PDB ID: 1A9U) in the benchmark datasets as the test case. We run all methods 3 trials and calculate the average time. The results are shown in Supplementary Table 1. Note that the time for each molecule counts the computationally expensive docking time. We can see the computational efficacy of ParetoDrug is similar to AlphaDrug but slower than the well-developed molecular design tool REINVENT 4. The reason may be that AlphaDrug and ParetoDrug use the additional pretrained model (i.e. Lmser Transformer) to guide molecule generation. But the pretrained model is necessary to help generate molecules with higher binding affinities.

Supplementary Table 1: Comparison of computational efficacy of RL or MCTS-based methods with $n = 3$ trails. The unit of Average Time and Time Per Molecule is second. The 95% confidence interval of mean values is given.

Method	Average Time	Average Number of Molecules	Time Per Molecule
AlphaDrug	16260±2598	1417±54	11.47
REINVENT 4	14420±178	2048±0	7.04
ParetoDrug	18040±2215	1498±115	12.04

D. Score Distributions of A Given Target

We also plot the generated molecules for a given target (PDB ID: 1A9U) by running AlphaDrug, ParetoDrug, and REINVENT 4. The running statistics are given in Appendix and the molecule score distributions are plotted in Supplementary Figure 1. Note that we plot the top 500 molecules (no repeated molecules) with the highest Docking scores collected in three trials for each method to reflect the score distribution of the algorithms. We can see that AlphaDrug has the best Docking score distribution but its LogP and QED distributions are in terrible situations. For example, as shown in Supplementary Figure 1, almost all the molecules generated by AlphaDrug have a QED value of less than 0.4 and the LogP values of most molecules are larger than the upper threshold (+5.6). Notably, ParetoDrug shifts the score distributions of AlphaDrug, which sacrifices the Docking score to achieve better QED and LogP in this case to generate molecules with balanced properties. Meanwhile, as REINVENT 4 does not belong to the target-aware molecule generation method, it has the lowest Docking score, which shows the importance of the pretrained molecule generative model which is conditioned on the target protein information. In conclusion, ParetoDrug tries to achieve a balance among these possibly conflicting properties. Nevertheless,

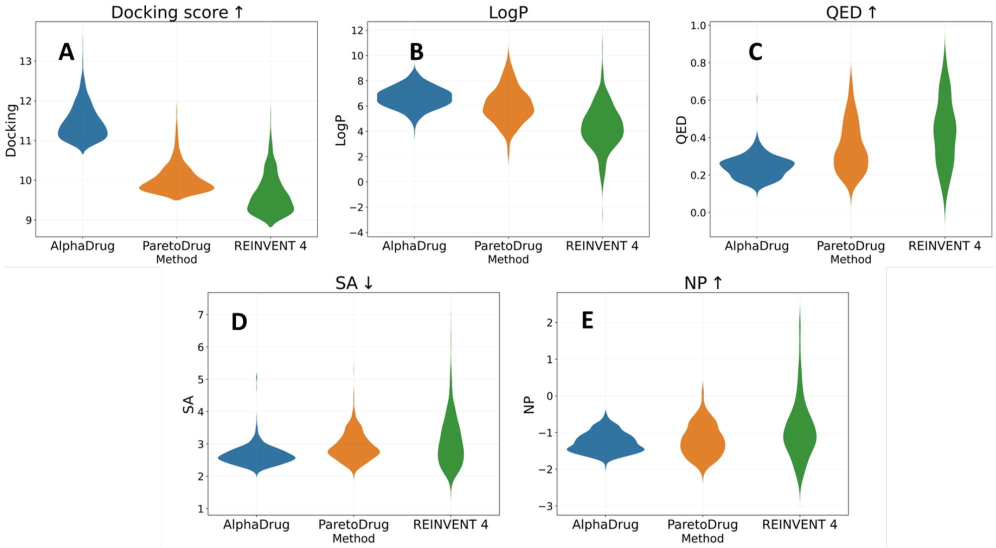

Supplementary Figure 1: Property distributions of generated molecules by AlphaDrug, ParetoDrug, and REINVENT 4 for a given target (PDB ID: 1A9U) with $n = 500$ molecules. (A) The docking score ($\text{kcal} \cdot \text{mol}^{-1}$) distributions of each method. (B) The LogP value distributions of each method. (C) The QED value distributions of each method. (D) The SA score distributions of each method. (E) The NP-likeness distributions of each method.

the score distributions indicate that ParetoDrug is exploring different chemical spaces with AlphaDrug although they utilize the same pretrained generative model.

E. Diversity of Generated Molecules

In this section, we study the molecule diversity of ParetoDrug. AlphaDrug and ParetoDrug perform MCTS with guidance from the pretrained generative model. The two algorithms explore during the MCTS by the selection randomness, which brings the molecule diversity. Specifically, the diversity of ParetoDrug is achieved by the selection randomness, which could be divided into two parts as indicated by Eq. (6) in the article. First, the pretrained model would provide the next-atom distribution based on the current inputting molecule fragment. This brings the randomness for generating molecules. This randomness is also for AlphaDrug. Second, when selecting the next node, ParetoDrug selects randomly from the Pareto-dominate candidate set. As there is no dominant molecule in most cases, different molecules would be generated for evaluation. This randomness is not for AlphaDrug.

We also perform experiments to examine the molecule diversity of ParetoDrug. We give the statistics of running 5 trials on the same target with AlphaDrug and ParetoDrug. The diversity ratio is calculated as the number of unique molecules (do not appear in the other four trials) in this trial divided by the number of molecules in the same trial. Results are shown in Supplementary Table 2. From Supplementary

Table 2, we found that most molecules generated in trial 2 and trial 3 of AlphaDrug are the same. ParetoDrug could have a better diversity ratio than AlphaDrug by the second selection randomness based on the Pareto-dominate candidate set. This is a desired property for molecule generation as we always want more new molecule candidates with good properties.

Supplementary Table 2: Diversity Ratio for Molecules in Each Trial.

Method	Trial 1	Trial 2	Trial 3	Trial 4	Trial 5
AlphaDrug	96.3%	21.0%	24.4%	68.1%	76.7%
ParetoDrug	81.1%	88.7%	86.8%	81.6%	85.2%

F. Protein-ligand Interaction Analysis

For the analysis of the protein-ligand interactions, we use PLIP[6], a fully automated protein-ligand interaction web tool. We mainly analyze two kinds of interactions, the hydrogen bond and the π-π stacking. The hydrogen bond is an attractive interaction between a hydrogen atom from a molecule or a molecular fragment X-H in which X is more electronegative than H, and an atom or a group of atoms in the same or another molecule, in which there is evidence of a bond formation[7]. The π-π stacking refers to the interaction between aromatic rings governed by an interplay of electrostatic, van-der-Waals, and hydrophobic interactions[8]. Stable arrangements of aromatic rings are either in parallel (sandwich) or a perpendicular (T-shaped) orientation[9]. Both the hydrogen bond and π-π stacking are recognized as the most important factors of the stabilization mechanism in the protein-ligand interaction analysis.

G. Settings of MM-GBSA

MM-GBSA is the end-point approach employing molecular mechanics, the generalized Born model, and solvent accessible surface area method to estimate binding free energies from structural information, overcoming the computational complexities associated with molecular simulations. Within the MM-GBSA approach, parameters are defined under the additivity approximation, treating the free energy change as a sum of various physical energy components.

We performed molecular dynamics simulations for all protein-ligand complexes using *pmemd.cuda* in the AMBER22 package[10] with the *ff19SB*[11] and *gaff* (version 1.81)[12] force fields respectively for the proteins and ligands. The HIS residue was protonated at the epsilon position. Partial charges of ligands are calculated using the AM1-BCC model[13]. The protein-ligand structures were solvated in the TIP3P[14] water box and the distance between the solute and the edge of the box was at least 10.0 Å. Na+ or Cl- ions were added to neutralize the unbalanced charge. The positions of added waters do not take into account the presence of the solute or ions. For more information, please refer to Supplementary Table 3. To reduce these bad contacts,

three energy minimization steps were preformed to create a stable system for the next production simulations. In the first step, the energy of water and ions was minimized and the protein backbone was restrained with an elastic constant of 50 kcal $\cdot$ mol$^{-1}\cdot$ Å^{-2}. In the second step, the elastic constant decreased to 10 kcal$\cdot$mol$^{-1}\cdot$ Å^{-2}. At last, the whole system was minimized without any restraint. The first 2000 cycles utilize the steepest descent algorithm before shifting to the conjugate gradient algorithm for the remaining 3000 cycles for every minimization. The cutoff for the non-bonded interactions (van der Waals and electrostatic interactions) was set to 10.0 Å. The particle mesh Ewald (PME)[15] algorithm was employed to estimate the long-range electrostatic interactions with the periodic boundary condition (PBC).

Then, during 50 ps, the minimized system was heated from 0 to 300 K in the NVT ensemble, followed by the 100-ps NPT equilibration runs at 1 atm pressure with weak restraints of 2.0 kcal $\cdot$ mol$^{-1}\cdot$ Å^{-2} on the heavy atoms of the proteins and 500-ps NPT equilibration runs without any restraint. In the last production runs, the conformations of protein-ligand complexes were sampled using 100 ns NPT molecular dynamics at 300 K with the Langevin thermostat and 1 atm pressure. The SHAKE[16] algorithm was used to constrain the covalent bonds involving hydrogen atoms. The time step was set to 2 fs and 20,000 snapshots were saved for every complex. For each system, three independent simulations were performed from the docked pose.

Time-course analysis of root mean square deviations (RMSDs) and Root mean square fluctuations (RMSFs) for all molecular simulations (shown in Supplementary Figures 2-5) was done using the *cpptraj* (version 4.14.0) module in AMBER18.

We uniformly extract 100 samples from the last 10,000 snapshots for MM-GBSA binding free energy calculations (shown in Supplementary Tables 4-7). Note that, in this study, a newer variant of MM-GBSA with the variable dielectric constant model implemented in a modified version of *MMPBSA.py* in AMBER18 was used[17]. The key idea is to assign the alternative dielectric value for atoms of the solute in the polar solvation free energies and electrostatic interactions described as

$$E_{\text{ele}} = \sum_k \frac{1}{\varepsilon_{\text{in}(k)}} \sum_{i \in k, j} \frac{q_i q_j}{r_{ij}} \tag{1}$$

and

$$E_{\text{GB}} = -\sum_k \left(\frac{1}{\varepsilon_{\text{in}(k)}} - \frac{1}{\varepsilon_{\text{sol}}}\right) \sum_{i \in k, j} \frac{q_i q_j}{\sqrt{r_{ij}^2 + \alpha_{ij}^2 \exp -(\frac{r_{ij}^2}{4\alpha_{ij}^2})}}, \tag{2}$$

where q_i is the charge on the atom i within the protein, and q_j is the charge within the atom j of the ligand, and r_{ij} is the distance between the atom i and atom j. The symbols ε_{in} and ε_{sol} correspond to the dielectric constants of the solute and the solvent (usually water) respectively. Meanwhile, α_{ij} is defined as the geometric average of the Born radii α_i and α_j. In the variable dielectric model, different dielectric constant ε_{in} is assigned to the residue type k, which interacts with the ligand.

Supplementary Table 3: The molecular dynamics condition for the complex of each compound and its protein target.

Ligand	Target	No. simulations	No. atoms	No. water	Box dimensions	No. salts
Tropifexor	FXR	3	32734	9587	65.517*81.908*76.502	10 Na+
Compound 1	FXR	3	32721	9587	65.517*81.908*76.502	10 Na+
Compound 2	FXR	3	32729	9587	65.517*81.908*76.503	10 Na+
Compound 3	FXR	3	32732	9587	65.517*81.908*76.504	10 Na+
Compound 4	FXR	3	32729	9587	65.517*81.908*76.502	10 Na+
Copanlisib	PI3K-γ	3	98370	27937	124.847*98.214*96.459	8 Na+
Compound 5	PI3K-γ	3	98359	27938	124.847*98.214*96.459	8 Na+
LigBuilder V3 de novo	HIV-PR	3	28535	8458	62.619*74.160*78.018	6 Cl-
LigBuilder V3 growing	HIV-PR	3	28532	8458	62.619*74.160*78.018	5 Cl-
LigBuilder V3 linking	HIV-PR	3	28543	8458	62.619*74.160*78.018	5 Cl-
Pocket2Mol-screen	HIV-PR	3	28532	8457	62.619*74.160*78.018	6 Cl-
TargetDiff-screen	HIV-PR	3	28539	8451	62.619*74.160*78.018	5 Cl-
Compound 6	HIV-PR	3	28551	8454	62.619*74.160*78.018	6 Cl-
Compound 7	HIV-PR	3	28546	8453	62.619*74.160*78.018	5 Cl-
Compound 8	HIV-PR	3	28548	8453	62.619*74.160*78.018	6 Cl-
LigBuilder V3 de novo	HIV-RT	3	144532	42833	113.958*112.893*133.143	6 Cl-
LigBuilder V3 growing	HIV-RT	3	144532	42834	113.958*112.893*133.143	5 Cl-
LigBuilder V3 linking	HIV-RT	3	144543	42834	113.958*112.893*133.143	5 Cl-
Pocket2Mol-screen	HIV-RT	3	144532	42833	113.958*112.893*133.143	6 Cl-
TargetDiff-screen	HIV-RT	3	144524	42822	113.958*112.893*133.143	5 Cl-
Compound 6	HIV-RT	3	144533	42824	113.958*112.893*133.143	6 Cl-
Compound 7	HIV-RT	3	144534	42825	113.958*112.893*133.143	5 Cl-
Compound 8	HIV-RT	3	144533	42824	113.958*112.893*133.143	6 Cl-

Supplementary Table 4: The energy terms in MM-GBSA for the complex of each compound and the protein target FXR (PDB ID: 5G2N).

Name	TRAJ	ΔVDW	ΔEEL	ΔEGB	ΔENP	ΔGGAS	ΔGSOL	ΔG
Tropifexor	1	-52.6741	28.2355	-21.8358	-6.7241	-24.4386	-28.5599	-52.9985
	2	-55.8346	22.8205	-15.7572	-7.2317	-33.0141	-22.9889	-56.0030
	3	-64.4776	29.5339	-21.4025	-8.0572	-34.9437	-29.4597	-64.4033
Compound 1	1	-49.1099	21.3140	-15.5012	-6.1126	-27.7959	-21.6138	-49.4097
	2	-56.9937	61.8665	-56.0014	-7.2851	4.8728	-63.2865	-58.4137
	3	-57.5891	18.7238	-11.4534	-7.1914	-38.8653	-18.6448	-57.5101
Compound 2	1	-58.9981	32.3734	-24.8113	-7.4709	-26.6246	-32.2822	-58.9068
	2	-59.0599	63.3978	-54.3439	-7.4081	4.3379	-61.7520	-57.4140
	3	-65.0134	39.4783	-31.0861	-7.9432	-25.5351	-39.0294	-64.5645
Compound 3	1	-58.3373	76.1604	-67.8224	-7.2999	17.8231	-75.1223	-57.2992
	2	-66.7320	113.0910	-102.8450	-8.4248	46.3590	-111.2700	-64.9112
	3	-66.8550	166.6012	-155.1410	-8.6792	99.7461	-163.8200	-64.0739
Compound 4	1	-59.5498	-35.3727	42.0782	-7.7601	-94.9225	34.3181	-60.6044
	2	-63.6409	5.1156	1.6317	-8.1780	-58.5254	-6.5463	-65.0716
	3	-66.2399	-24.0845	29.8994	-8.3735	-90.3244	21.5259	-68.7985

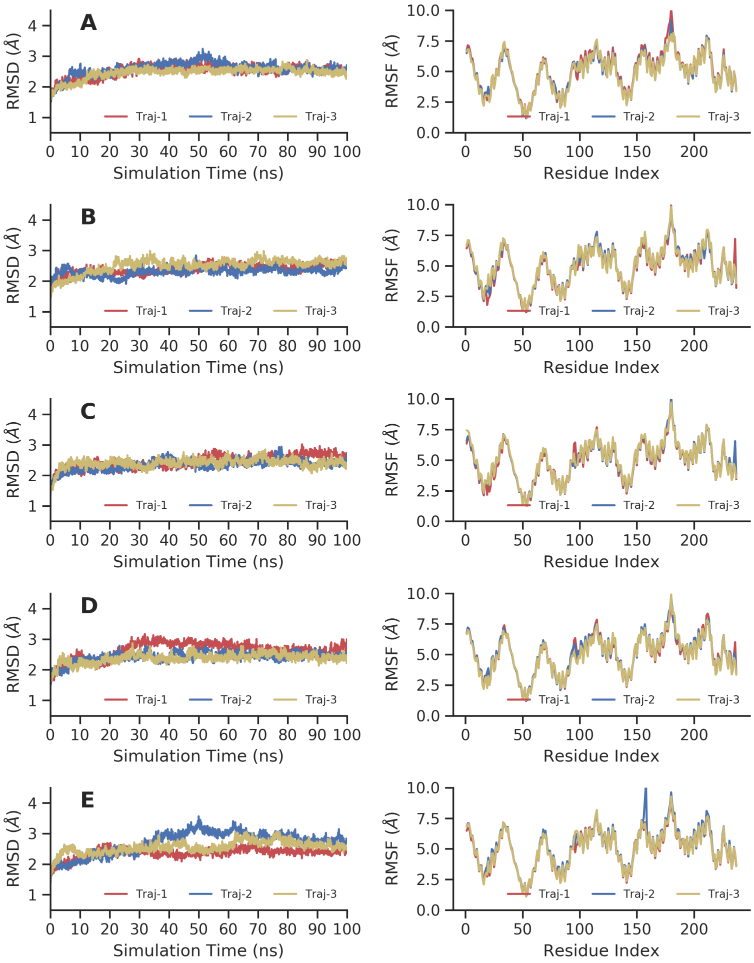

Supplementary Figure 2: Time course of RMSDs and RMSFs for the complexes of molecules binding to the protein target FXR (PDB ID: 7D42). (A) RMSD (*left*) and RMSF (*right*) of Tropifexor binding to FXR. (B) RMSD and RMSF of Compound 1 binding to FXR. (C) RMSD and RMSF of Compound 2 binding to FXR. (D) RMSD and RMSF of Compound 3 binding to FXR. (E) RMSD and RMSF of Compound 4 binding to FXR.

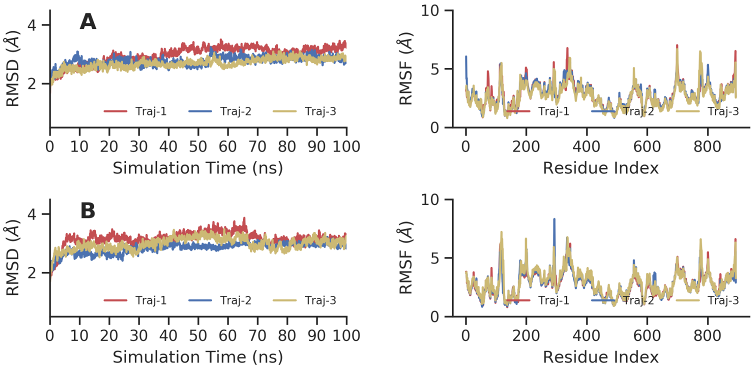

Supplementary Figure 3: Time course of RMSDs and RMSFs for the complexes of molecules binding to the protein target PI3K-γ (PDB ID: 5G2N). (A) RMSD (*left*) and RMSF (*right*) of Tropifexor binding to PI3K-γ. (B) RMSD and RMSF of Compound 5 binding to PI3K-γ.

Supplementary Table 5: The energy terms in MM-GBSA for the complex of each compound and the protein target PI3K-γ (PDB ID: 7D42).

Name	TRAJ	ΔVDW	ΔEEL	ΔEGB	ΔENP	ΔGGAS	ΔGSOL	ΔG
	1	-56.1286	75.4198	-66.9233	-6.5191	19.2912	-73.4425	-54.1513
Copanlisib	2	-57.7872	-114.7660	124.2624	-6.9036	-172.5540	117.3587	-55.1948
	3	-58.7285	55.4279	-47.1937	-6.6936	-3.3006	-53.8873	-57.1879
	1	-41.3045	73.0538	-67.8416	-5.5630	31.7492	-73.4045	-41.6553
Compound 5	2	-45.2515	-16.0000	20.8389	-6.1418	-61.2514	14.6971	-46.5544
	3	-49.5740	-17.3996	22.1165	-6.3674	-66.9736	15.7491	-51.2244

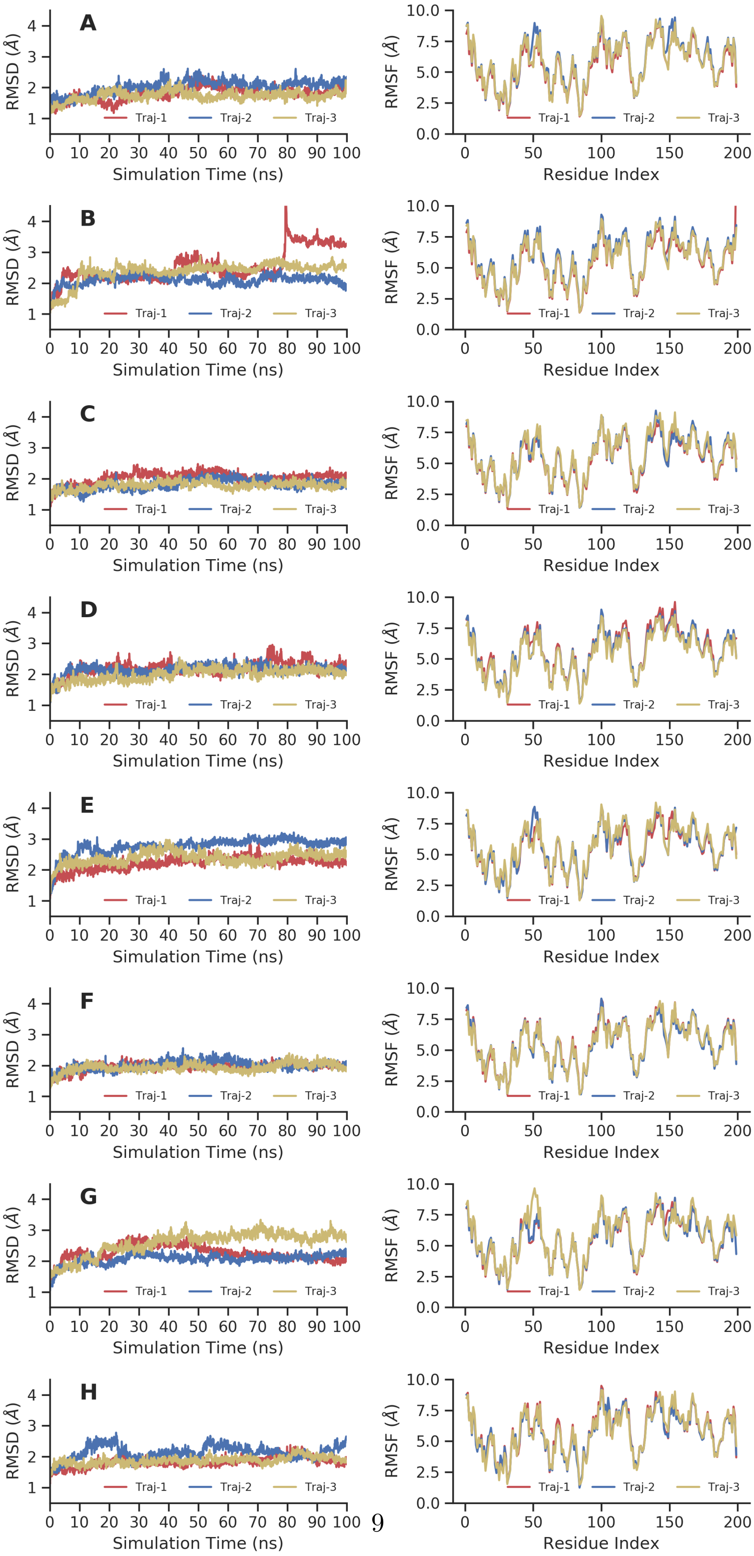

Supplementary Figure 4: Time course of RMSDs and RMSFs for the complexes of molecules binding to the protein target HIV-PR (PDB ID: 3A2O). (A) RMSD (*left*) and RMSF (*right*) of molecule from LigBuilder V3 de novo binding to HIV-PR. (B) RMSD and RMSF of molecule from LigBuilder V3 growing binding to HIV-PR. (C) RMSD and RMSF of molecule from LigBuilder V3 linking binding to HIV-PR. (D) RMSD and RMSF of molecule from Pocket2Mol-screen binding to HIV-PR. (E) RMSD and RMSF of molecule from TargetDiff-screen binding to HIV-PR. (F) RMSD and RMSF of Compound 6 binding to HIV-PR. (E) RMSD and RMSF of Compound 6 binding to HIV-PR. (E) RMSD and RMSF of Compound 6 binding to HIV-PR.

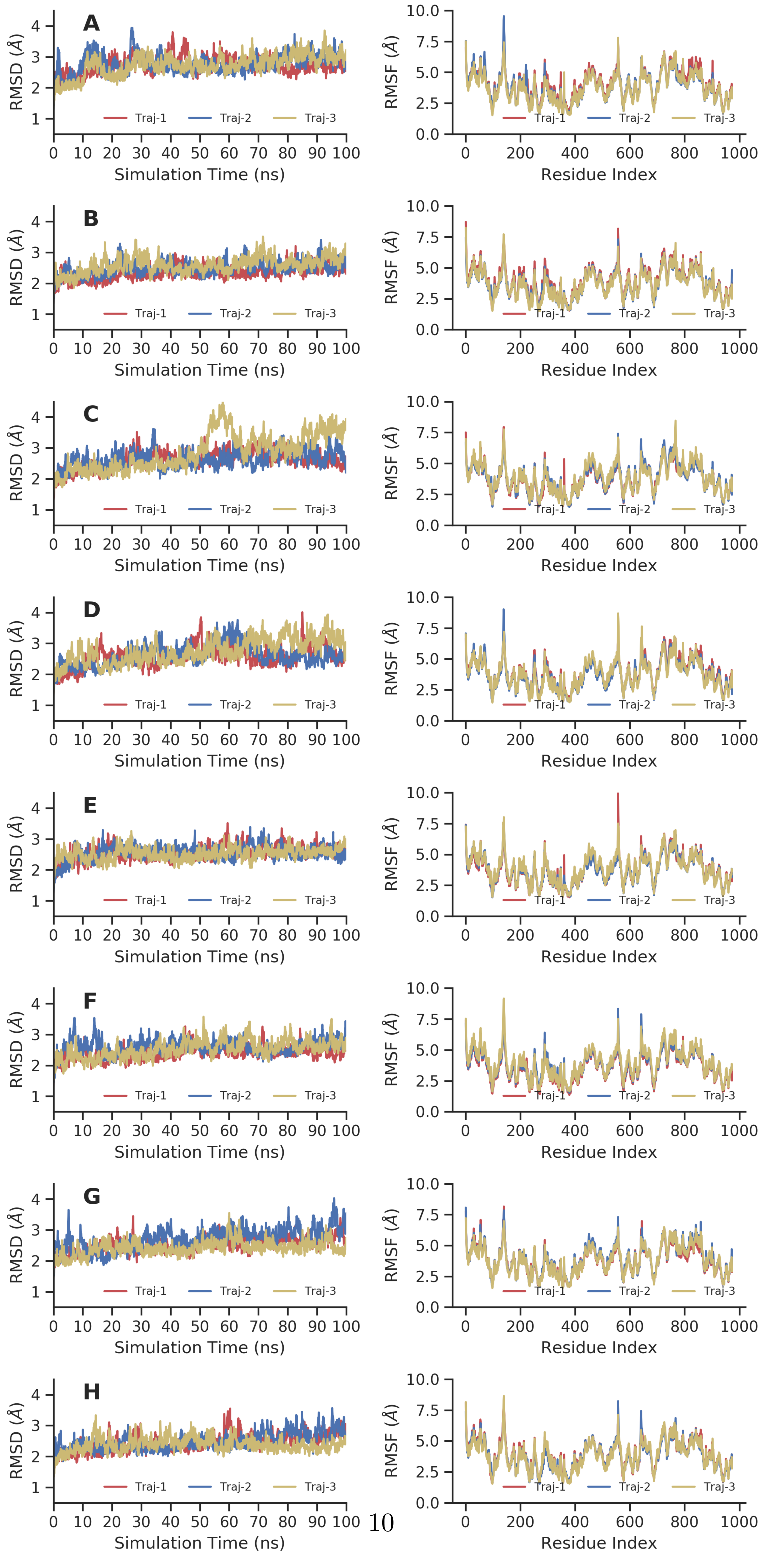

Supplementary Figure 5: Time course of RMSDs and RMSFs for the complexes of molecules binding to the protein target HIV-RT (PDB ID: 4G1Q). (A) RMSD (*left*) and RMSF (*right*) of molecule from LigBuilder V3 de novo binding to HIV-RT. (B) RMSD and RMSF of molecule from LigBuilder V3 growing binding to HIV-RT. (C) RMSD and RMSF of molecule from LigBuilder V3 linking binding to HIV-RT. (D) RMSD and RMSF of molecule from Pocket2Mol-screen binding to HIV-RT. (E) RMSD and RMSF of molecule from TargetDiff-screen binding to HIV-RT. (F) RMSD and RMSF of Compound 6 binding to HIV-RT. (E) RMSD and RMSF of Compound 6 binding to HIV-RT. (E) RMSD and RMSF of Compound 6 binding to HIV-RT.

Supplementary Table 6: The energy terms in MM-GBSA for the complex of each compound and the protein target HIV-PR (PDB ID: 3A2O).

Name	TRAJ	ΔVDW	ΔEEL	ΔEGB	ΔENP	ΔGGAS	ΔGSOL	ΔG
LigBuilder V3 de novo	1	-23.9705	-75.6517	77.9435	-3.1122	-99.6222	74.8313	-24.7909
	2	-33.2366	-47.3607	51.7531	-4.0660	-80.5973	47.6872	-32.9101
	3	-33.4216	-14.7498	18.6160	-3.8931	-48.1714	14.7229	-33.4485
LigBuilder V3 growing	1	-18.5446	-23.9989	25.2930	-2.0652	-42.5435	23.2278	-19.3157
	2	-25.5332	-11.6140	13.8476	-3.0213	-37.1472	10.8263	-26.3209
	3	-28.1972	-62.3932	63.9167	-3.2298	-90.5904	60.6869	-29.9035
LigBuilder V3 linking	1	-35.0602	-19.3285	22.6382	-3.9192	-54.3887	18.7190	-35.6697
	2	-38.0866	-78.0848	81.4891	-4.0211	-116.1710	77.4679	-38.7035
	3	-38.9169	-76.9145	80.3789	-4.2981	-115.8310	76.0809	-39.7505
Pocket2Mol-screen	1	-17.8042	-0.3972	1.5675	-1.5252	-18.2013	0.0423	-18.1590
	2	-36.4298	7.1087	-5.1292	-2.9726	-29.3211	-8.1019	-37.4229
	3	-48.0696	-5.9551	8.3339	-3.8129	-54.0247	4.5210	-49.5037
TargetDiff-screen	1	-28.4842	-65.0559	67.2576	-2.4362	-93.5401	64.8214	-28.7187
	2	-48.9462	-57.1128	62.8538	-4.8018	-106.0590	58.0520	-48.0070
	3	-55.4434	-81.6987	86.2138	-6.1066	-137.1420	80.1072	-57.0348
Compound 6	1	-61.4959	3.3924	3.4856	-6.7269	-58.1035	-3.2413	-61.3448
	2	-67.7715	40.7527	-33.7564	-6.9896	-27.0188	-40.7460	-67.7648
	3	-74.1741	-69.5831	77.3630	-7.8422	-143.757	69.5209	-74.2363
Compound 7	1	-51.4346	46.4022	-41.3029	-5.1266	-5.0324	-46.4295	-51.4619
	2	-64.6469	34.3281	-28.4200	-5.5144	-30.3187	-33.9344	-64.2531
	3	-67.0714	-88.5985	93.2797	-6.0104	-155.6700	87.2694	-68.4006
Compound 8	1	-46.9553	56.6613	-51.8698	-4.9899	9.7061	-56.8597	-47.1536
	2	-55.3722	-42.9760	48.6678	-5.9659	-98.3483	42.7020	-55.6463
	3	-60.2755	-2.5639	11.0553	-6.3340	-62.8394	4.7213	-58.1181

Supplementary Table 7: The energy terms in MM-GBSA for the complex of each compound and the protein target HIV-RT (PDB ID: 4G1Q).

Name	TRAJ	ΔVDW	ΔEEL	ΔEGB	ΔENP	ΔGGAS	ΔGSOL	ΔG
	1	-45.1487	48.5428	-43.6471	-4.6493	3.3941	-48.2964	-44.9023
LigBuilder V3 de novo	2	-48.5218	52.3198	-48.4103	-4.4010	3.7980	-52.8113	-49.0134
	3	-48.8967	3.5711	0.3054	-4.5263	-45.3255	-4.2209	-49.5464
	1	-27.0488	-122.2040	124.3068	-3.7527	-149.2530	120.5541	-28.6985
LigBuilder V3 growing	2	-41.5636	-58.6782	62.0025	-5.1423	-100.2420	56.8602	-43.3815
	3	-43.8816	-68.2716	72.2239	-4.8278	-112.1530	67.3961	-44.7571
	1	-58.9644	-103.2000	110.0372	-6.4200	-162.1640	103.6172	-58.5467
LigBuilder V3 linking	2	-59.9053	-128.0920	135.6068	-6.9088	-187.9980	128.6980	-59.2995
	3	-61.6788	-165.8170	173.9206	-6.6598	-227.4950	167.2608	-60.2346
	1	-57.2264	13.3653	-10.8346	-4.8556	-43.8611	-15.6902	-59.5513
Pocket2Mol-screen	2	-60.0658	15.4612	-12.1341	-4.6018	-44.6046	-16.7359	-61.3405
	3	-60.2899	4.2398	-1.0726	-4.5881	-56.0502	-5.6606	-61.7108
	1	-61.1948	-215.8180	222.0484	-5.7522	-277.0130	216.2962	-60.7170
TargetDiff-screen	2	-66.7885	-36.7802	42.2940	-6.0833	-103.5690	36.2106	-67.3581
	3	-69.9391	-138.4720	144.2658	-6.8624	-208.4110	137.4035	-71.0076
	1	-79.9117	143.3755	-132.4830	-7.7399	63.4638	-140.2230	-76.7593
Compound 6	2	-80.8827	-25.3865	38.2028	-8.0415	-106.2690	30.1614	-76.1079
	3	-84.1863	29.2493	-20.1556	-7.5721	-54.9371	-27.7276	-82.6647
	1	-105.477	-33.9309	44.9410	-9.1691	-139.4080	35.7719	-103.636
Compound 7	2	-81.6694	267.1939	-253.9700	-7.4814	185.5244	-261.4520	-75.9272
	3	-82.9564	231.6384	-220.5750	-7.4341	148.6820	-228.0090	-79.3270
	1	-76.3881	24.8390	-14.6941	-7.5036	-51.5491	-22.1978	-73.7468
Compound 8	2	-83.7372	161.6071	-150.0490	-8.0555	77.8699	-158.1050	-80.2346
	3	-83.9414	99.3793	-85.2479	-7.8929	15.4379	-93.1408	-77.7029

Supplementary References

[1] Qian, H., Lin, C., Zhao, D., Tu, S., Xu, L.: AlphaDrug: protein target specific de novo molecular generation. PNAS Nexus **1**(4), 227 (2022)

[2] Liu, T., Lin, Y., Wen, X., Jorissen, R.N., Gilson, M.K.: BindingDB: a web-accessible database of experimentally determined protein-ligand binding affinities. Nucleic Acids Research **35**(Database), 198–201 (2007)

[3] Grechishnikova, D.: Transformer neural network for protein-specific de novo drug generation as a machine translation problem. Scientific Reports **11**(1), 321 (2021)

[4] Koes, D.R., Baumgartner, M.P., Camacho, C.J.: Lessons Learned in Empirical Scoring with smina from the CSAR 2011 Benchmarking Exercise. Journal of Chemical Information and Modeling **53**(8), 1893–1904 (2013)

[5] Berman, H.M.: The Protein Data Bank. Nucleic Acids Research **28**(1), 235–242 (2000)

[6] Salentin, S., Schreiber, S., Haupt, V.J., Adasme, M.F., Schroeder, M.: PLIP: fully automated protein–ligand interaction profiler. Nucleic Acids Research **43**(W1), 443–447 (2015)

[7] Arunan, E., Desiraju, G.R., Klein, R.A., Sadlej, J., Scheiner, S., Alkorta, I., Clary, D.C., Crabtree, R.H., Dannenberg, J.J., Hobza, P., Kjaergaard, H.G., Legon, A.C., Mennucci, B., Nesbitt, D.J.: Definition of the hydrogen bond (IUPAC Recommendations 2011). Pure and Applied Chemistry **83**(8), 1637–1641 (2011)

[8] Salentin, S., Haupt, V.J., Daminelli, S., Schroeder, M.: Polypharmacology rescored: Protein–ligand interaction profiles for remote binding site similarity assessment. Progress in Biophysics and Molecular Biology **116**(2-3), 174–186 (2014)

[9] Müller-Dethlefs, K., Hobza, P.: Noncovalent Interactions: A Challenge for Experiment and Theory. Chemical Reviews **100**(1), 143–168 (2000)

[10] Case, D.A., Aktulga, H.M., Belfon, K., Cerutti, D.S., Cisneros, G.A., Cruzeiro, V.W.D., Forouzesh, N., Giese, T.J., Götz, A.W., Gohlke, H., Izadi, S., Kasavajhala, K., Kaymak, M.C., King, E., Kurtzman, T., Lee, T.-S., Li, P., Liu, J., Luchko, T., Luo, R., Manathunga, M., Machado, M.R., Nguyen, H.M., O'Hearn, K.A., Onufriev, A.V., Pan, F., Pantano, S., Qi, R., Rahnamoun, A., Risheh, A., Schott-Verdugo, S., Shajan, A., Swails, J., Wang, J., Wei, H., Wu, X., Wu, Y., Zhang, S., Zhao, S., Zhu, Q., Cheatham, T.E., Roe, D.R., Roitberg, A., Simmerling, C., York, D.M., Nagan, M.C., Merz, K.M.: AmberTools. Journal of Chemical Information and Modeling **63**(20), 6183–6191 (2023)

[11] Tian, C., Kasavajhala, K., Belfon, K.A.A., Raguette, L., Huang, H., Migues,

A.N., Bickel, J., Wang, Y., Pincay, J., Wu, Q., Simmerling, C.: ff19SB: Amino-Acid-Specific Protein Backbone Parameters Trained against Quantum Mechanics Energy Surfaces in Solution. Journal of Chemical Theory and Computation **16**(1), 528–552 (2020)

[12] Wang, J., Wolf, R.M., Caldwell, J.W., Kollman, P.A., Case, D.A.: Development and testing of a general amber force field. Journal of Computational Chemistry **25**(9), 1157–1174 (2004)

[13] Jakalian, A., Jack, D.B., Bayly, C.I.: Fast, efficient generation of high-quality atomic charges. AM1-BCC model: II. Parameterization and validation. Journal of Computational Chemistry **23**(16), 1623–1641 (2002)

[14] Jorgensen, W.L., Chandrasekhar, J., Madura, J.D., Impey, R.W., Klein, M.L.: Comparison of simple potential functions for simulating liquid water. The Journal of Chemical Physics **79**(2), 926–935 (1983)

[15] Darden, T., York, D., Pedersen, L.: Particle mesh Ewald: An N.log(N) method for Ewald sums in large systems. The Journal of Chemical Physics **98**(12), 10089–10092 (1993)

[16] Ryckaert, J.-P., Ciccotti, G., Berendsen, H.J.C.: Numerical integration of the cartesian equations of motion of a system with constraints: molecular dynamics of n-alkanes. Journal of Computational Physics **23**(3), 327–341 (1977)

[17] Wang, E., Fu, W., Jiang, D., Sun, H., Wang, J., Zhang, X., Weng, G., Liu, H., Tao, P., Hou, T.: VAD-MM/GBSA: A Variable Atomic Dielectric MM/GBSA Model for Improved Accuracy in Protein–Ligand Binding Free Energy Calculations. Journal of Chemical Information and Modeling **61**(6), 2844–2856 (2021)
